# Computational Prediction of Coiled–Coil Protein Gelation Dynamics and Structure

**DOI:** 10.1021/acs.biomac.3c00968

**Published:** 2023-12-18

**Authors:** Dustin Britton, Luc F. Christians, Chengliang Liu, Jakub Legocki, Yingxin Xiao, Michael Meleties, Lin Yang, Michael Cammer, Sihan Jia, Zihan Zhang, Farbod Mahmoudinobar, Zuzanna Kowalski, P. Douglas Renfrew, Richard Bonneau, Darrin J. Pochan, Alexander J. Pak, Jin Kim Montclare

**Affiliations:** † Department of Chemical and Biomolecular Engineering, New York University Tandon School of Engineering, Brooklyn, New York 11201, United States; ‡ Department of Chemical and Biological Engineering, Colorado School of Mines, Golden, Colorado 80401, United States; § National Synchrotron Light Source-II, Brookhaven National Laboratory, Upton, New York 11973, United States; ∥ Microscopy Laboratory, New York University Langone Health, New York, New York 10016, United States; ⊥ Department of Materials Science and Engineering, University of Delaware, Newark, Delaware 19716, United States; # Center for Computational Biology, Flatiron Institute, Simons Foundation, New York, New York 10010, United States; ¶ Center for Genomics and Systems Biology, New York University, New York, New York 10003, United States; ∇ Courant Institute of Mathematical Sciences, Computer Science Department, New York University, New York, New York 10009, United States; ○ Quantitative Biosciences and Engineering, Colorado School of Mines, Golden, Colorado 80401, United States; ⧫ Department of Chemistry, New York University, New York, New York 10012, United States; †† Department of Biomedical Engineering, New York University, New York, New York 11201, United States; ‡‡ Bernard and Irene Schwartz Center for Biomedical Imaging, Department of Radiology, New York University School of Medicine, New York, New York 10016, United States; §§ Department of Biomaterials, New York University College of Dentistry, New York, New York 10010, United States

## Abstract

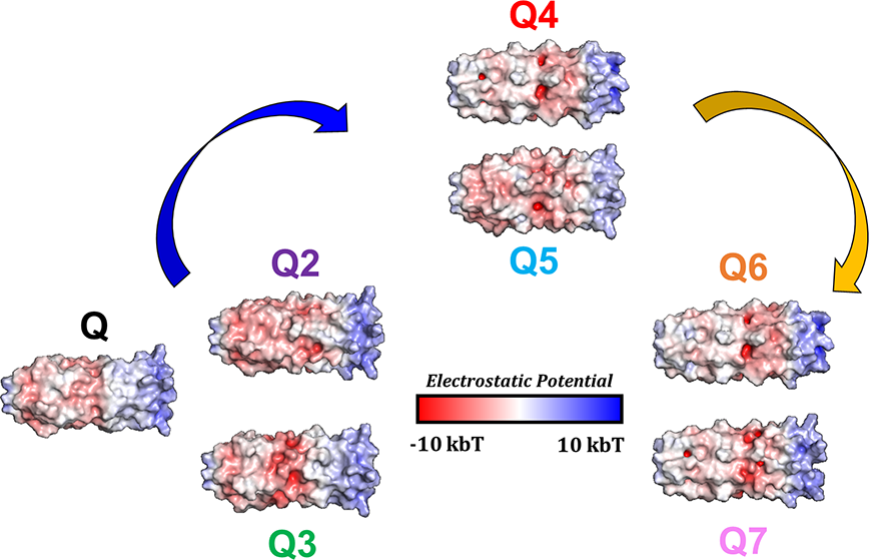

Protein hydrogels represent an important and growing biomaterial for a multitude of applications, including diagnostics and drug delivery. We have previously explored the ability to engineer the thermoresponsive supramolecular assembly of coiled–coil proteins into hydrogels with varying gelation properties, where we have defined important parameters in the coiled–coil hydrogel design. Using Rosetta energy scores and Poisson–Boltzmann electrostatic energies, we iterate a computational design strategy to predict the gelation of coiled–coil proteins while simultaneously exploring five new coiled–coil protein hydrogel sequences. Provided this library, we explore the impact of in silico energies on structure and gelation kinetics, where we also reveal a range of blue autofluorescence that enables hydrogel disassembly and recovery. As a result of this library, we identify the new coiled–coil hydrogel sequence, Q5, capable of gelation within 24 h at 4 °C, a more than 2-fold increase over that of our previous iteration Q2. The fast gelation time of Q5 enables the assessment of structural transition in real time using small-angle X-ray scattering (SAXS) that is correlated to coarse-grained and atomistic molecular dynamics simulations revealing the supramolecular assembling behavior of coiled–coils toward nanofiber assembly and gelation. This work represents the first system of hydrogels with predictable self-assembly, autofluorescent capability, and a molecular model of coiled–coil fiber formation.

## Introduction

Protein-based hydrogels, particularly those that are stimuli-responsive, are increasingly explored as biomaterials for diagnostics and drug delivery.^1^ Compared to synthetic-based polymers, proteins benefit from a high modularity enabled by the large number of sequence permutations derived from the 20 canonical amino acids and a growing non-canonical amino acid library.^1,2^ These numerous sequence possibilities lead to specific protein folds and structures including α-helical coiled–coils, which in the case of hydrogels, lead to a variety of assemblies and nonassemblies.^3^ Despite the large compositional space of protein sequences, conferring gelation properties through predictive tuning of a protein sequence remains untapped, with most design relegated to domain-by-domain approaches thus far.^4^ Creating protein biomaterials with a variety of hydrogel properties would allow for the design of protein-based hydrogels with desired properties, such as the control of gelation time. This would enable application-specific designs where hydrogels predicted to possess slow gelation times would be well-suited for tissue engineering^5^ and hydrogels designed for fast gelation times, <1 h, would be clinically considered for in situ gelation for drug delivery.^6^

Computational design of protein hydrogels has largely used modeling and simulation ex post facto.^1^ Current computational workflows mostly reside in the simulation space where the ability to represent and model self-assembly of known sequences compared to a complete library of sequence combinations^7,8^ or a small library of rational mutants^9^ are the dominant approaches. Conversely, computationally driven designs have not been well-explored and those that have must still be screened using molecular dynamics (MD) simulations that come at a high computational cost.^10^ Developing quantitative metrics to correlate the sequence directly to the hydrogel function would help to avoid these pitfalls and bridge the gap between sequence and function. Thus, most protein-based hydrogels have existed in the realm of naturally occurring and recombinant protein fusions.^1^ These hydrogels include lower critical solution temperature (LCST) hydrogels such as elastin-like polypeptides (ELPs)^10−12^ and silk peptide fusions such as silk-elastin like peptides (SELPs).^13,14^

Previously, we have designed upper critical solution temperature (UCST) coiled–coil protein hydrogels by targeting electrostatic interactions between the N-terminus possessing a positively charged surface patch and the C-terminus possessing a negatively charged surface patch.^15,16^ Notably, significant differences in physically cross-linked fiber diameters and their respective critical gelation times were reported for proteins designed to possess a lower electrostatic potential and improved thermostability.^16^ While protein domain folding^16−18^ or MD software^19^ has been used to assess stability of coiled–coil self-assembly, a broader gap exists in sequence-structure-gelation relationships. Recently, we have established a quantitative relationship between fiber diameters and the electrostatic potential difference between the N- and C- termini of surface residues in the b, c, and f helical wheel positions (ΔEE_bcf_) as a metric to understand the role of electrostatic interactions on supramolecular nanofiber assembly.^17^

Herein, ΔEE_bcf_ is used to study fiber diameter and gelation as supramolecular assembly properties. Furthermore, ΔEE_bcf_ is used to expand our library of coiled–coil hydrogels by characterizing five newly generated hydrogel sequences. In doing so, an iterative workflow is used based on Rosetta energy scores and ΔEE_bcf_ to generate new sequences consecutively and to define regression models for prediction of coiled–coil gelation times. Strong relationships among Rosetta energy scores, thermoresponsiveness, fluorescent recovery after photobleaching (FRAP), and structural transitions during gelation are established. In the case of FRAP, the wide variety of fluorescence behavior and hydrogel recovery after induced fluorescence introduces a new inherent signal of coiled–coil hydrogels.

Among the coiled–coil hydrogel sequences studied, Q5 undergoes a complete sol–gel transition within 24 h, possessing a critical gelation time, *t*_c_, of 11.5 h, a greater than 2-fold increase to previously reported coiled–coiled hydrogels.^16^ For fiber formation, transmission electron microscopy (TEM) suggests a mechanism predominantly dependent on end-to-end stacking.^20^ The fast gelation time allows the structural changes within a full 24 h window to be assessed using small-angle X-ray scattering (SAXS) measurements to elucidate the interparticle (interchain) interaction and supramolecular assembly of the coiled–coil hydrogel. The interchain interactions and supramolecular assemblies are further studied by using coarse-grained (CG) MD simulations via measurements of radial density distributions and supramolecular structures across fibril sizes and effective electrostatic interaction strengths.

## Materials and Methods

### Materials

Chemically competent M15MA *E. coli* cells were gifted from David Tirrell at California Institute of Technology.^21^ Bacto-tryptone, sodium chloride (NaCl), yeast extract, tryptic soy agar, ampicillin sodium salt, sodium phosphate dibasic anhydrous (Na_2_HPO_4_), sodium hydroxide (NaOH), dextrose monohydrate (d-glucose), magnesium sulfate (MgSO_4_), calcium chloride (CaCl_2_), manganese chloride tetrahydrate (MnCl_2_·4H_2_O), cobaltous chloride hexahydrate (CoCl_2_·6H_2_O), isopropyl β-d-1-thiogalactopyranoside (IPTG), Pierce bicinchoninic acid (BCA) assay kit, Pierce snakeskin dialysis tubing 3.5k molecular weight cutoff (MWCO), sodium dodecyl sulfate (SDS), Nunc ninety-six well plates, Molecular Probes FluoSpheres (1.0 μm), phosphotungstic acid, and BD Clay Adams glass microscopy slides were acquired from Thermo Fisher Scientific. The 20 naturally occurring amino acids and thiamine hydrochloride (vitamin B) were purchased from Sigma-Aldrich. Hydrochloric acid (HCl) and Coomassie Brilliant Blue G-250 were purchased from VWR. HiTrap FF 5 mL columns for protein purification were purchased from Cytiva Life Sciences. Macrosep and Microsep Advance Centrifugal Devices 3k molecular weight cutoff (MWCO) and 0.2 μm syringe filters were purchased from PALL. Acrylamide/bis solution (30%) 29:1, and natural polypeptide sodium dodecyl sulfate–polyacrylamide gel electrophoresis (SDS–PAGE) standard were purchased from Bio-Rad. Imidazole was purchased from Acros Organics. Formvar/carbon-coated copper grids (FCF400-Cu) and 1% uranyl acetate for transmission electron microscopy were purchased from Electron Microscopy Sciences. Borosilicate glass capillaries (0.2 × 2 × 75 mm) were purchased from VitroCom. Fast-curing two-component epoxy was acquired from JB Weld.

### Computational Design of Variant Library

Rosetta suite of macromolecular modeling tools (Version 3.5) was used to model protein mutants and calculate Rosetta scores, with lower energy scores indicating higher stability. The Rosetta Relax protocol^22^ was used on protein sequences maintaining the symmetry of COMPcc (PDB: 3V2P) with the all-atom energy score function.^23^ Q2 and Q3 were designed previously,^17^ where Q2 has been characterized as a hydrogel and fiber^16,17^ and Q3 as a fiber.^17^ PDB2QR and APBS^24^ were used to calculate the electrostatic potential of surface residues, EE_bcf_, for the N- and C-termini. The difference of the N- and C-terminal EE_bcf_, dubbed ΔEE_bcf_, was calculated as described previously^17^ (eq 1).
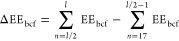
1

The Q2 sequence was used as an initial input sequence for the iterative mutation into sequences Q4 and Q5. Iterative mutations were made through a Monte Carlo search repeated 60 times for 1000 iterations with the goal of minimizing the Rosetta score. The Monte Carlo search used eq 2 to determine the probability of selecting a mutant with a worse (higher) Rosetta score (*P*_RS_)
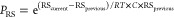
2where RS is the Rosetta score [J/mol], *RT* [J/mol] is the product of the molar gas constant and temperature, and *C* is an empirical constant used to constrain the probability criteria during the search (a *C* value of 3.93 × 10^–5^ [mol/J] was used in our searches). Sequences with the lowest Rosetta scores from each Monte Carlo search were aggregated into a list, and residues with the highest likelihood of occurrence at each position were used in the final sequences for Q4 and Q5. Probability plots were made using WebLogo (version 2.8.2).

The critical gelation times, *t*_c_, of Q, Q2, Q4, and Q5—variants soluble at 2 mM for microrheology measurements—were plotted against ΔEE_bcf_, with the variants possessing ΔEE_bcf_ values of approximately 31, 41, 45, and 64 × 10^4^ kJ/mol, respectively. To improve regression model confidence and expand the library of gelation times achieved, ΔEE_bcf_ values between 45 and 64 × 10^4^ kJ/mol were targeted. Thus, a trimodal Monte Carlo search targeting an improved (1) Rosetta score, (2) EE_bcf_ at the N-terminus (NEE_bcf_), and (3) EE_bcf_ at the C-terminus (CEE_bcf_) were used to acquire sequences for Q6 and Q7 where an improved Rosetta score is a lower value and an improved NEE_bcf_ and CEE_bcf_ are closer in value to the target NEE_bcf_ and CEE_bcf_. NEE_bcf_ and CEE_bcf_ values of approximately 27 × 10^4^ and 80 × 10^4^ kJ/mol were used as target values to acquire a ΔEE_bcf_ in between 45 and 64 × 10^4^ kJ/mol, respectively, while possessing an improved Rosetta score. Equation 3 was used to determine the probability of selecting a worse EE_bcf_(*P*_EE_bcf__) at the N- or C- terminus
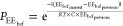
3where a *C* of 1.31 × 10^–4^ [mol/J] and 1.96 × 10^–4^ [mol/J] was used in eq 3 in our searches for N- and C-terminal EE_bcf_, respectively. The resulting Q6 gel possessed a ΔEE_bcf_ of 51 × 10^4^ kJ/mol. Similarly, after characterizing the *t*_c_ of Q6, a sequence Q7 was selected from the previous search results based on its low Rosetta score and ΔEE_bcf_ existing between 51 × 10^4^ and 64 × 10^4^ kJ/mol. Final protein structures were visualized using PyMOL^25^ with the APBS plugin.^24^

### Protein Expression

All Q variants were expressed as described previously using supplemented M9 media grown from 16 mL starter cultures (Supporting Information).^15^

### Protein Purification

All Q variants were purified using previously described purification protocols by lysing cells with a Q500 probe sonicator (QSonica), removing cell debris by centrifugation and purifying via a cobalt-charged HiTRAP IMAC FF 5 mL column using an increasing gradient of imidazole (Supporting Information).^15^

### Rheological Assessment

Rheology with a parallel plate geometry was used to assess mechanical strength and microrheology was used to assess gelation kinetics of Q hydrogel variants, as has been done for Q2 protein previously (Supporting Information).^15,16^

### Tube Inversion

Binary sol–gel behavior was assessed using tube inversion.^16^ Immediately after concentration to 2 mM, 150 μL of Q hydrogel variants were incubated in 2 mL microtubes at 4 °C for 2 weeks. Gelation was visually observed by inverting the microtube and inspecting the sample for flow from the top of the tube where weak or strong gel-like behavior was considered a gel.

### Secondary Structure Assessment

Secondary structure and deconvolution of pre- and postgelation Q variants were assessed by circular dichroism (CD) spectra on a Jasco J-815 CD spectrometer and attenuated total reflectance-Fourier transform infrared (ATR-FTIR) spectra on a Nicolet 6700 Fourier Transform Infrared Spectrometer equipped with a mercury cadmium telluride (MCT)-A detector as described previously (Supporting Information).^26,27^

### Transmission Electron Microscopy

Transmission electron microscopy (TEM) images were taken with a FEI Talos L120C transmission electron microscope as described previously (Supporting Information).^15^

### Confocal Imaging and FRAP

FRAP experiments were performed with a Stellaris 8 Falcon laser scanning confocal microscope on an inverted DMi8 CS stand equipped with a 63× NA 1.4 lens. Images were taken with a laser with 405 nm excitation. A prebleach image was taken followed by two bleach images using 100% laser intensity for 1.5 s. Following, 41 images were taken postbleach at 2 s intervals. To measure the recovery, FIJI (version 2.3.0) was used to automate background and recovery imaging following photobleaching. Independent background intensities were fit to a one-phase decay equation in GraphPad Prism. Background loss, as a result of photobleaching, from fit values was added to spot intensity values before being fit to a one-phase association equation in GraphPad Prism.

### Small Angle X-ray Scattering

SAXS was employed to measure interparticle interactions, structure, and shape of Q5 since it was capable of gelation in the instrument holder within 24 h. SAXS experiments were performed at the National Synchrotron Light Source-II (NSLS-II) Beamline 16-ID (LiX) at the Brookhaven National Laboratory (Supporting Information).

### Cryo-Electron Microscopy

Lacey carbon 300 mesh copper grids (Ted Pella) were used for cryo-EM imaging on FEI TALOS F200C with a Ceta 16 M camera at 200 kV accelerating voltage. PELCO easiGlow Glow Discharge Cleaning System was also used for the plasma treatment of the grids (Supporting Information).

### Statistical Analysis

GraphPad Prism (GraphPad Software) was employed for statistical analysis using a Student’s *t*-test.

### All-Atom Molecular Dynamics

The atomic structure of pentameric Q5 (referred to as CC hereafter) was generated using Alphafold2Multimer^28^ (version 2.1.2) using the full database and maximum template date of 01/01/2018. Based on the pLDDT confidence metric predicted by Alphafold2 (Table S5), as well as the known pentameric COMPcc channel from which Q was originally derived, the pentamer form of Q5 was deemed the most likely. Two CCs (i.e., 10 Q5 monomers) were stacked end-to-end separated by 6 nm. The two CCs were set up in a 20 × 10 × 10 nm^3^ box with periodic boundary conditions and solvated by 0.5 M NaCl in water. The simulation was run using the CHARMM36m force field (February 2021) and GROMACS 2021.1.^29,30^ Initially, energy minimization was done using the steepest descent with a tolerance of 500 kJ/mol/nm. Simulations were equilibrated first in the canonical (*NVT*) ensemble to 277 K (4 °C) for 0.5 ns using the stochastic velocity rescaling thermostat and a damping time of 0.1 ps.^31^ Next, the system was equilibrated to 1 bar under the *NPT* ensemble for 1 ns using the Parrinello–Rahman barostat and a damping time of 20 ps.^32^ Finally, simulations were run in the *NVT* ensemble for 1.2 μs at 277 K (4 °C) using stochastic velocity rescaling and a damping time of 2 ps;^31^ only the final 1.1 μs of data was used for analysis with coordinates saved every 100 ps. For all simulations, a 2 fs time step was used, and the α carbons of residues 14 to 39 of the lower CC were harmonically restrained with a force constant of 1000 kJ/mol/nm^2^. All simulations were repeated across four independent replicas.

### Coarse-Grained Model

CG sites were mapped to the α-carbon positions of each residue such that each CG site represented one amino acid. Two sets of virtual sites were also mapped: intra-CC virtual sites and inter-CC virtual sites.^33,34^ Ten intra-CC virtual sites were defined between CG sites for residues 16, 18, 21, 22, 25, 28, 31, 33, 36, and 38 in adjacent monomers to account for the anisotropic knob-in-hole interactions that maintain the CC structure. One inter-CC virtual site was defined between residues 12 and 50 at the end-to-end interface to account for end-to-end CC interactions. Four types of potentials defined our CG Hamiltonian: bonded, electrostatic, excluded volume, and virtual site attractions. All intramolecular nonbonded interactions and bonds were defined using a heteroelastic network model (HENM)^35^ with a cutoff of 19.0 Å using the CG-mapped atomistic trajectories with virtual sites added. Models were constructed using the OpenMSCG (v0.7.1) Python package.^36^ Complete details on CG model construction and parametrization are shown in the Supporting Information.

### Coarse-Grained Simulations

Fibril CG MD simulations were run using LAMMPS (2 Jun 2022).^37^ Fibril simulations were prepared in three stages: protofibril geometry optimization, multiprotofibril configurational optimization, and fibril dynamics (Figure S19). First, the energy of a CC pentamer was minimized using the conjugate gradient algorithm and a tolerance of 10^–6^ kcal/mol/Å. Subsequent protofibrils were built from the geometry-optimized CC structure. Then, multiple CCs were set up to approximate the arrangements within protofibrils and between protofibrils that yielded the lowest potential energies. To optimize the separation distance between CCs within a protofibril, an end-to-end stack of five CCs, i.e., a protofibril of length five, was constructed. The center-of-mass distance between CCs was uniformly adjusted between 56 and 75 Å to find the inter-CC spacing with minimum energy (Figure S16). This distance was used to construct all of the subsequent protofibrils. To optimize the fibril geometry, the previously optimized protofibrils were aligned in a square lattice with separation distances between 20 and 40 while adjacent protofibrils were staggered along the protofibril axis direction for distances between 0 and 60 Å. The energy of each configuration was computed to find the radial and staggered distances that yielded the lowest energy (Figure S16); these two distances were used to prepare the initial configurations of fibrils in the next stage. Finally, CG MD fibril simulations were prepared using a square lattice structure of *N* × *N* staggered protofibrils of length 14 CCs where *N* was varied from 2 to 10 protofibrils in increments of 2. For each fibril size, a dielectric constant of 10, 20, 30, 40, 45, 50, 60, 70, or 80 was used. Each system was equilibrated for 10 ns with a time step of 10 fs using the Nosé–Hoover chain thermostat^38^ at 277 K and 1 ps damping time. Trajectories were extended for 2 ns, and data was collected every 2 ps. All simulations were repeated across three independent replicas.

### Radial Density Distributions

The normalized radial density distribution  for each fibril (Figure S16) was computed using eq 4
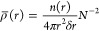
4where *r* is the pair distance, *n*(*r*) is the ensemble-averaged number of particles in the range *r* to *r* + δ*r*, δ*r* is the bin width (=0.5 Å), and *N* is the fibril size (e.g., *N* = 4 for the 4 × 4 fibril). The distribution function was computed between all CG sites (i.e., α carbon resolution) with all intracoiled–coil CG pairs subtracted to focus only on intercoiled–coil CG pairs. Another distribution function using only the centers-of-mass (COM) of each coiled–coil was computed; this distribution was multiplied by a factor of 20,000 such that the magnitude would be comparable to the α carbon distribution for visualization purposes.

### Phase Diagram

To estimate fibril diameters, we translated the fibril to the origin and rotated it to align the first two principal axes (computed based on the COM of each CC) to the *x*- and *z*-axis, respectively, such that the long direction of the fibril was aligned with the *x*-axis. This operation was performed on each frame of the trajectory. Next, protofibril-normalized COM histograms in the *y* and *z* directions were computed by using a bin width of 1 nm (Figure S18). The difference between the lower (*r*_lo_) and upper (*r*_hi_) bounds where the COM histogram exceeded 3.0 #/protofibril^1/2^ was used to define the normalized fibril diameter  (in nm/protofibril) (eq 5)
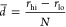
5

If no lower and upper bounds were detected, the average diameter was treated as 0 nm, i.e., the fibril dissociated. All analysis was done using the Numpy and MDTraj Python packages.^39^

## Results and Discussion

### Hydrogel Design

**Figure 1:**
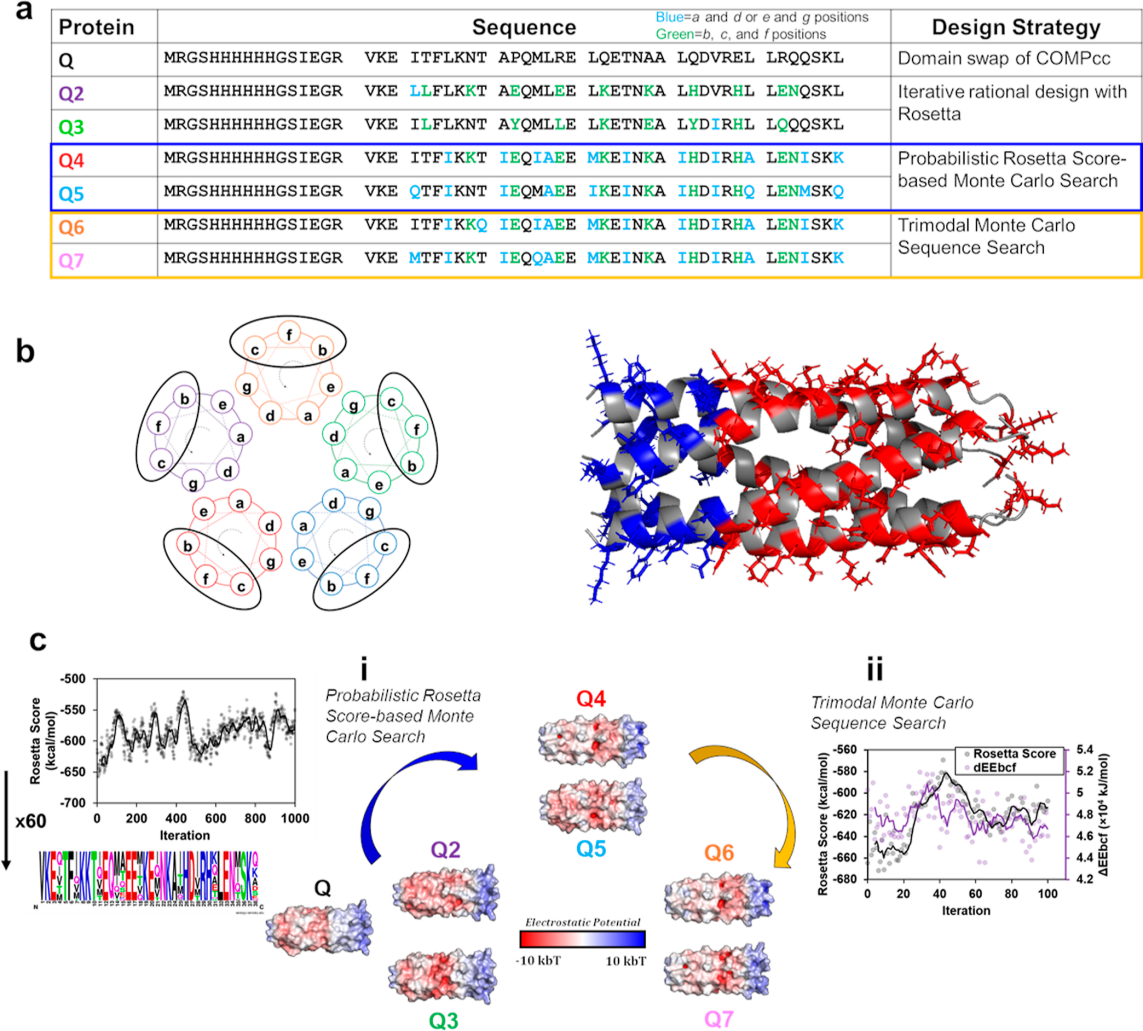
(a) Q hydrogel variant protein sequences with blue mutations highlighting differences made to the hydrophobic domain and green mutations highlighting differences to the coiled–coil surface compared to Q. (b) Helical wheel diagram of a pentamer with helical wheel positions (b–f) circled showing their surface location. PyMOL cartoon of Q5 with N-terminal residues, which contribute to a positive surface patch, shown in blue, and C-terminal residues, which contribute to a negative patch, shown in red. (c) Q hydrogel variant design order in which (i) probabilistic Rosetta score-based Monte Carlo Searches were aggregated to generate sequences for Q4 and Q5 where an example of the Q5 sequence probability plot is shown and (ii) a trimodal Monte Carlo search employing criteria for Rosetta score, NEE_bcf_, and CEE_bcf_ were used to find a targeted ΔEE_bcf_ for Q6 and Q7 sequences.

Hydrogel variants characterized herein are the result of previous rational design, (Q3), probabilistic Rosetta-based Monte Carlo searches (Q4 and Q5), and trimodal—Rosetta energy-based, NEE_bcf_, and CEE_bcf_—Monte Carlo searches (Q6 and Q7) (Figure 1a) where numbering is the order of the sequence design/characterization. Q and its sequel Q2 have been previously designed and characterized as hydrogel materials.^16^ We set out to automate the design process of coiled–coil hydrogels using Monte Carlo search algorithms and incorporating ΔEE_bcf_. Previously, we have established a proportional relationship between the coiled–coil fiber diameter and ΔEE_bcf_, where ΔEE_bcf_ represents the electrostatic potential difference of residues located on the surface of a coiled–coil at the b, c, and f helical wheel positions between the N-terminus (NEE_bcf_) and C-terminus CEE_bcf_^17^ (Figure 1b). As another metric, the Rosetta energy score represented the most thermodynamically stable conformations of the hydrogel variants after modeling in Rosetta^23^ and thus were used to compare relative coiled–coil stability. With only three variants capable of gelation, of which only Q and Q2 were soluble at high concentrations (≥2 mM), a Monte Carlo search is employed to obtain variants with an improved Rosetta score compared to Q, Q2, and Q3. In this search, the Q2 sequence is used as an input sequence and allowed to be mutated at positions in the a, d, e, and g positions; this is done to generate variants that possess similar surface charge distribution as well as solubility and thus an increased likelihood of gelation [Figure 1c(i)]. The resulting sequences, Q4 and Q5, are used to generate a linear model using ΔEE_bcf_ to predict the critical gelation time, *t*_c_. The model indicates a missing compositional space for hydrogels with *t*_c_ values between 30 and 70 h corresponding to ΔEE_bcf_ values between 45 and 64 × 10^4^ kJ/mol, respectively. We set out to fill this compositional space and improve the automation of our design process using the ΔEE_bcf_ metric. Using a trimodal Monte Carlo search, we target N- and C- terminal EE_bcf_ values to generate Q6 and Q7 to fill this compositional space [Figure 1c(ii)].

### Hydrogel Assembly and Prediction

**Figure 2:**
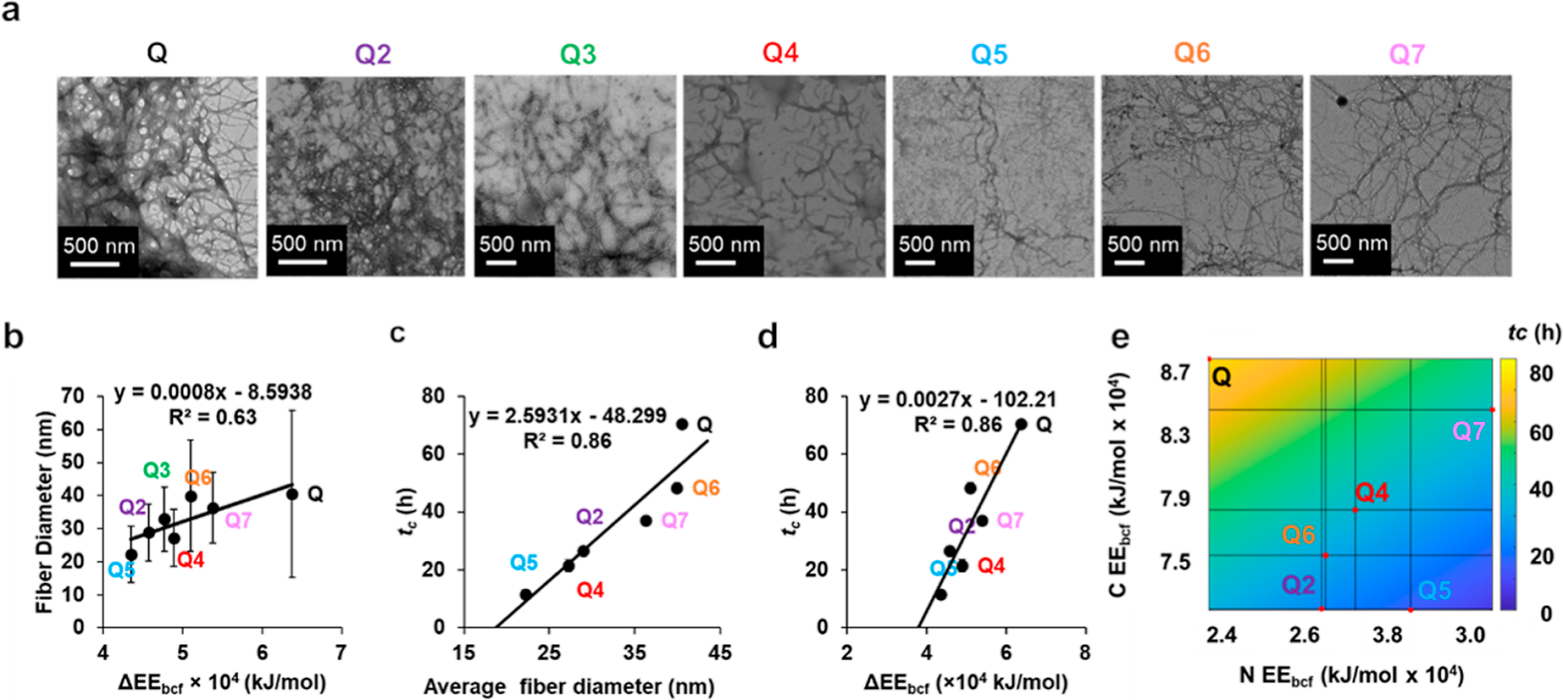
(a) TEM images of Q and Q2-7 with a scale bar at 500 nm. (b) Average nanofiber diameters by TEM for Q hydrogel variants (*n* = 100) and its correlation to ΔEE_bcf_ where error bars represent the standard deviation. (c) Linear relationship between the average fiber diameter and *t*_c_. (d) Linear relationship between *t*_c_ and ΔEE_bcf_. (e) Bivariate linear regression model for all Q hydrogel variants for NEE_bcf_, CEE_bcf_, and *t*_c_.

Q3, Q4, Q5, Q6, and Q7 were successfully expressed and purified as previously described^16^ (Figures S1–S5). Gelation was assessed by concentrating protein variants to 2 mM (1.3% w/v) in tris buffer (50 mM tris, 500 mM NaCl) at pH 8.0 within 8 h of the starting concentration. Hydrogel assembly was first assessed using TEM images of the physically cross-linked nanofibers after incubation at 4 °C (Figure 2a) and samples were visually inspected for gelation by tube inversion. Independent representative fibers (*n* = 100) were identified and measured from images of the Q variants to determine average differences in lateral assembly, which were also compared to previously characterized Q and Q2 hydrogel nanofibers.^16^ The average protein fiber diameters were ranked from smallest to largest with 22.2 ± 8.4 nm for Q5, 27.2 ± 8.7 nm for Q4, 28.9 ± 8.6 nm for Q2,^16^ 32.8 ± 9.8 nm for Q3, 36.3 ± 10.8 nm for Q7, 39.9 ± 16.9 nm for Q6, and 40.5 ± 25.2 nm for Q.^16^ By an unpaired *t*-test, all fiber populations were determined to be significantly different, except for the following pairs and *p*-values: Q2/Q4: 0.1662, Q6/Q7: 0.0742, Q6/Q: 0.8434, Q7/Q: 0.1271 (Figure S6). Consistent with previously measured differences of the nanofiber library, Q fibers were significantly larger than Q3 and Q2 fibers.^17^ Moreover, the growth in the hydrogel fiber diameter also possesses a strong linear relationship with ΔEE_bcf_ (Figure 2b). Notably, this relationship was weaker than the linear relationship for the large nanofibers synthesized under denaturing conditions found previously, which exhibited *R*^2^ = 0.95.^17^ This was partially attributed to the physical entanglement of hydrogels, leading to the presence of isolated fibers in addition to fiber bundles, resulting in proportionally higher standard deviations and lower ranges of hydrogel fiber diameters. Another factor contributing to the variability in the fiber diameter could be the collapse of fibers during the sample drying process on the grids. This phenomenon has been substantiated through a comparison of Q5 fiber diameters in cryo-electron microscopy.

Gelation kinetics of the hydrogels was assessed using passive microrheology (Figure S7a–e). Time to gelation was measured immediately after concentration to 2 mM at 4 °C by incubating with 1 μm fluorescent tracer beads added to the protein in solution state at a final concentration of 1% (v/v). Microrheology at 2 mM was performed for all variants with the exception of Q3, which possessed a solubility limit of approximately 1.5 mM, and was thus left out of analyses comparing gelation kinetics and mechanical strength. Mean square displacements (MSDs) of bead trajectories were analyzed periodically per the span of respective protein gelation times. An initial MSD–τ curve corresponding to a solution at 1.00 μm^2^ s^–1^ (consistent with Brownian motion) was used as the master solution curve, and the final MSD–τ curve measured per protein gelation was selected as the master gel curve. Intermediate MSD–τ curves measured were superimposed onto either the master solution or master gel curve by using horizontal and vertical shift factors, *a* and *b*, respectively. The window of sol–gel transition was determined between where a divergence in superposition occurred for each protein gelation.

The *t*_c_ is used to describe the time until the protein exhibits a majority gel behavior where tracer movement is confined rather than solution behavior where tracers exhibit Brownian motion. Both *t*_c_ and critical relaxation exponent, *n*_c_, are determined as previously described^16^ (Table S1). The *t*_c_ ranked in increasing order is 11.5 ± 1.5 h for Q5, 21.6 ± 2 h for Q4, 26.6 ± 0.5 h for Q2,^16^ 37.1 ± 0.1 h for Q7, 48.3 ± 1.7 h for Q6, 58 ± 0.4 h for Q3, and 70.4 ± 0.1 h for Q.^15^ The *n*_c_ is typically used to assess the degree of cross-linking. The *n*_c_ for the new protein hydrogels are ranked in increasing order with 0.56 ± 0.03 for Q4, 0.49 ± 0.02 for Q5, 0.60 ± 0.03 for Q6, and 0.58 ± 0.00 for Q7, suggesting the gels to be at the boundary of a loosely cross-linked hydrogel,^40^ similar to Q^15^ and Q2.^16^ We employ *t*_c_ to explore the relationship with fiber diameter, as suggested by the faster gelation times of thinner Q2 fibers compared to Q. *t*_c_ exhibits a strongly positive linear relationship with an average fiber diameter (Figure 2c), which, in turn reveals a strong relationship with ΔEE_bcf_ (Figure 2d), indicating the ability to predict gelation by linear regression. This relationship is also consistent with our previous finding that reduced lateral assembly of fibers may lead to a higher density of cross-linking and thus faster gelation kinetics.^16^

After the design and characterization of Q4 and Q5 hydrogels, we used a preliminary linear relationship to correlate ΔEE_bcf_ to *t*_c_ (Figure S8) as an early iteration to predict and design future variants. Gelation times of 36 and 44 h were predicted for Q6 and Q7, respectively, with a root mean squared error (RMSE) of 5.5 h and *R*^2^ = 0.95 based on this linear relationship where Q6 and Q7 were measured to possess gelation times of ∼48 and ∼37 h, respectively. The final model (Figure 2d) possessed an RMSE of 13.0 h and an *R*^2^ = 0.86.

With the six variants measured for gelation time at 2 mM, we have expanded this model further by delineating the weight of N- and C- terminal EE_bcf_ and EE_bcf_ (NEE_bcf_ and CEE_bcf_, respectively) and generating a bivariate linear relationship (eq 6, Figure 2e).
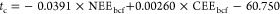
6

This relationship increases the correlation slightly from *R*^2^ = 0.86 to *R*^2^ = 0.88 and helps to explain the relative contribution of the N- and C-termini to *t*_c_. The RMSE is also reduced to 6.9 h (Figure S9). Contribution from the N-terminus shown here is due to five surface residues (AA17-28) in these protein variants, whereas the C-terminus contribution is the result of 11 surface residues (AA29-54). As a result, the N-terminus is weighted more strongly in the gelation prediction and thus self-assembly of our Q hydrogel variants with a coefficient of −7.82 × 10^–4^ h mol kJ^–1^ AA^–1^, whereas the C-terminus coefficient is 2.36 × 10^–4^ h mol kJ^–1^ AA^–1^, approximately 3-fold greater. Thus, mutations of the N-terminus of the coiled–coil region have a relatively higher impact on the self-assembly and gelation of Q hydrogel variants.

### Thermoresponsiveness and Material Strength

**Figure 3:**
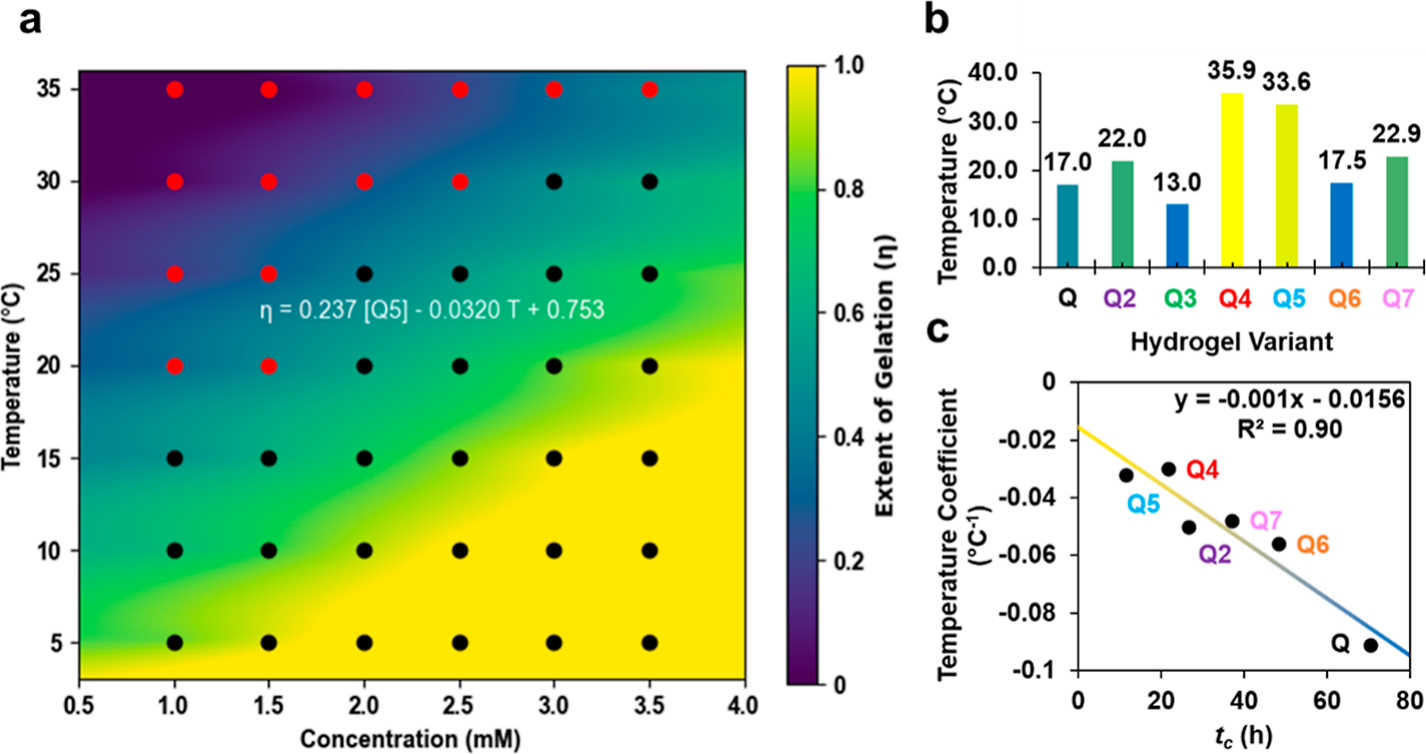
(a) Q5 extent of gelation, η, calculated by bivariate linear regression of a concentration–temperature phase diagram constructed from tube inversions where black markers represent gel behavior and red markers represent solution behavior after 2 weeks of incubation at 4 °C. η is defined as the phase equilibrium between solution-like and gel-like hydrogel behavior. (b) Maximum upper critical solution temperature measured at the solubility limit for Q and Q2^16^ and Q3–7. (c) Linear correlation of the temperature (dependence) coefficient calculated from respective phase diagrams and *t*_c_ where the temperature coefficient is defined as the independent linear dependence calculated using the bivariate linear regression of η in a and Figure S10a–d.

UCST behavior of protein hydrogels was assessed by testing 100 mL samples via tube inversion in 2 mL microtubes after incubation for 2 weeks at various temperatures and concentrations. Temperature conditions were modulated in the range of 5–40 °C. The concentration range was modified depending on if precipitation was visually noticeable in the microtube at an earlier concentration, indicating a solubility limit to our phase diagram, as has been done previously.^16,17^ These solubility limits for Q and Q2-7 were found to be 3.5 3.5, 1.5, 4.0, 3.5, 3.0, and 3.0 mM, respectively. As done with Q2, we determined the UCST of the hydrogels using a bivariate linear relationship for the extent of gelation where tubes that passed gelation by tube inversion were assigned a value of 1 (shown in black in Figures 3a and S10a–d) and tubes that failed gelation by tube inversion (shown in red in Figures 3a and S10a–d) were assigned a value of 0. The heatmap in Figures 3a and S10a–d was used to denote the range of extent of gelation where the equation was employed to solve for the UCST using the solubility limit and solving for an extent of gelation, η, value of 0.5. The UCSTs (Figure 3b) for the hydrogels studied here were 13.0, 35.9, 33.6, 17.5, and 22.9 °C for Q3-7, respectively, as compared to 17.0 °C for Q and 22.0 °C for Q2 found previously.^16^ Bivariate regression allowed for solving the coefficients for the dependence of the temperature and concentration on gelation. Interestingly, the dependence on temperature was strongly correlated (*R*^2^ = 0.90) with the critical gelation time, *t*_c_ (Figure 3c), suggesting that the thermostability of the hydrogel was indicative of its gelation kinetics. Due to the significant time investment in periodic measurements during microrheology and large sample volumes required for full phase diagram measurements, this result suggested that carefully selected tube inversion testing of hydrogels for UCST may offer a less hands-on method for screening targeted coiled–coil gelation times, whereas microrheological assays may offer a lower production method to screen for targeted thermoresponsiveness.

**Figure 4:**
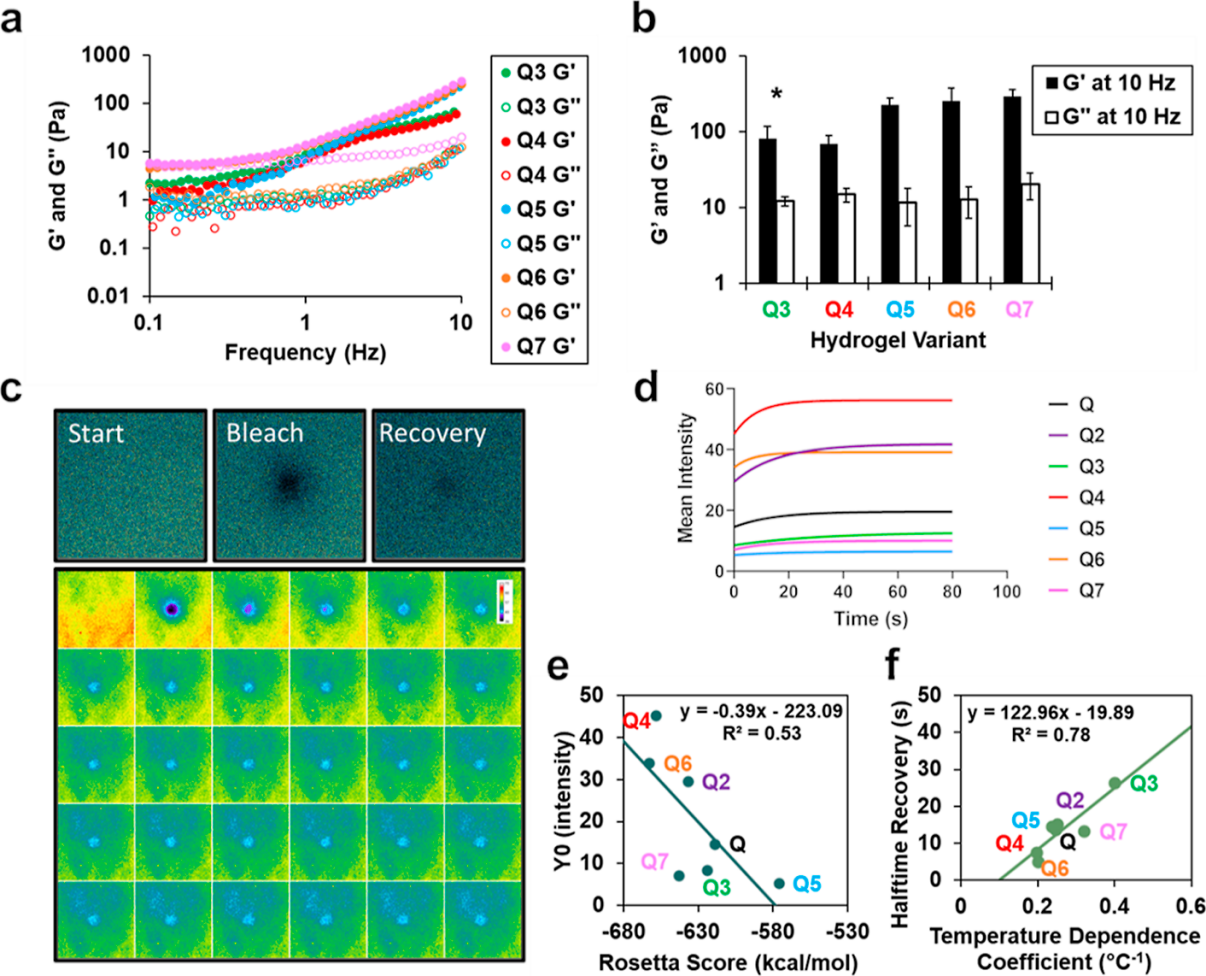
(a) Average storage modulus (*G*′, filled markers) and loss modulus (*G*″, empty markers) of Q3-7 measured using a parallel plate rheometer at various frequencies and (b) at 10 Hz. Error bars represent the standard deviation of three independent trials. * Q3 has a slightly lower concentration of 1.5 mM due to insolubility. (c) Representative FRAP imaging of the start (prebleach), bleach, and recovery (final postbleach) and corresponding montage showing the prebleach and the first 29 frames postbleach. (d) FRAP recovery curves were made by fitting to a one-phase association equation in Graphpad. (e) Linear correlation of Rosetta score to *Y*0 in one-phase association equation fit in Graphpad and (f) linear correlation of temperature dependence coefficient solved in respective phase diagrams to halftime recovery in one-phase association equation fit in Graphpad.

Following gelation, the storage (*G*′) and loss (*G*″) moduli of protein hydrogels were determined by rheological analysis (Table S1). Frequency sweeps were performed from 0.1 to 10 Hz at an oscillation strain of 5% (Figure 4a), with *G*′ and *G*″ at 10 Hz used to compare relative mechanical integrity of the variants (Figure 4b). Important to note, all variants were assessed at 2 mM while Q3 was assessed at 1.5 mM due to its relatively lower solubility limit compared to the other variants. The storage moduli of the hydrogels at 10 Hz were measured to be 83 ± 35 Pa for Q3, 70 ± 20 Pa for Q4, 229 ± 52 Pa for Q5, 253 ± 123 Pa for Q6, and 298 ± 62 Pa for Q7. Loss moduli at 10 Hz were measured to be 12 ± 2 Pa for Q3, 19 ± 4 Pa for Q4, 12 ± 6 Pa for Q5, 13 ± 6 Pa for Q6, and 21 ± 8 Pa for Q7, indicating that *G*′ > *G*″ for all hydrogel variants. All hydrogel variants assessed have a higher storage modulus than Q (50 Pa).^15^ Differences between the gels with the highest storage moduli (Q5, Q6, and Q7) were not statistically significant in comparison with those of the highest performer, Q2. Using this macroscopic assessment, we determine that Q2, Q5, Q6, and Q7 hydrogels have stronger networks than those of Q and Q4.

Protein fibrils are capable of blue autofluorescence as a result of protein self-assembly.^41^ Fluorescent recovery after photobleaching (FRAP) is measured by photobleaching using a 405 nm laser and measuring the spot intensity over time. We have previously confirmed this behavior in our Q protein in a recent characterization.^18^ Using the correlation between autofluorescence and fibrilization, we measured the relative autofluorescence of our coiled–coil protein hydrogels. Interestingly, the fluorescence intensities of the hydrogels diminished nearest the center of the laser, indicating a loss of fibrilization over time. To assess the relative laser response, hydrogels were subjected to FRAP imaging using two 100% intensity bleaches at 405 nm excitation followed by imaging at 2 s frame intervals (Figure 4c).

On average, hydrogels exhibited a loss of 67, 64, 64, 75, 68, 87, and 62% for Q–Q7, respectively, indicating that Q4 and Q6 experienced more resilience to bleaching. Recovery of the hydrogels over time was fit to a one-phase association equation in GraphPad Prism (Figure 4d and Table S2). Similarly, the hydrogels experienced an average recovery (in 80 s) to 88, 90, 92, 93, 83, 99, and 87% for Q–Q7, respectively. All hydrogels showed high recovery, especially Q6 with virtually complete recovery of its fluorescence intensity. The hydrogels also displayed varying relative fluorescence pre- and postbleaching, *Y*0, after baselining with background and applying the one-phase association equation fit. As expected, start intensities (Figure S11) and *Y*0 values were strongly correlated with *R*^2^ = 0.97. The *Y*0 values of the hydrogels possessed a positive correlation with their Rosetta energy scores (Figure 4e). The Rosetta energy scores measured here were of the pentameric coiled–coil symmetry and described the lowest pose energy found for a single coiled–coil. Fibril autofluorescence of the Q hydrogels was dependent on the self-assembly of the coiled–coil, whereas we have found that other gelation properties of the Q hydrogels, such as storage modulus and critical gelation time, were independent of Rosetta score.

The time to recovery, measured by the half–time parameter in the one-phase association equation in GraphPad Prism, possesses a strong correlation to the temperature dependence coefficient (Figure 4f) measured in the hydrogel phase diagrams (Figures 3a and S10a–d). The strong correlation (*R*^2^ = 0.79) indicates that bleaching of the hydrogels is similar to its temperature responsiveness and an entropic response to a high intensity laser. This also suggests the use of photoexcitation as a stimulus in future explorations of Q hydrogels for triggered drug delivery.

### Coiled–Coil Structure and Impact of Gelation

**Figure 5:**
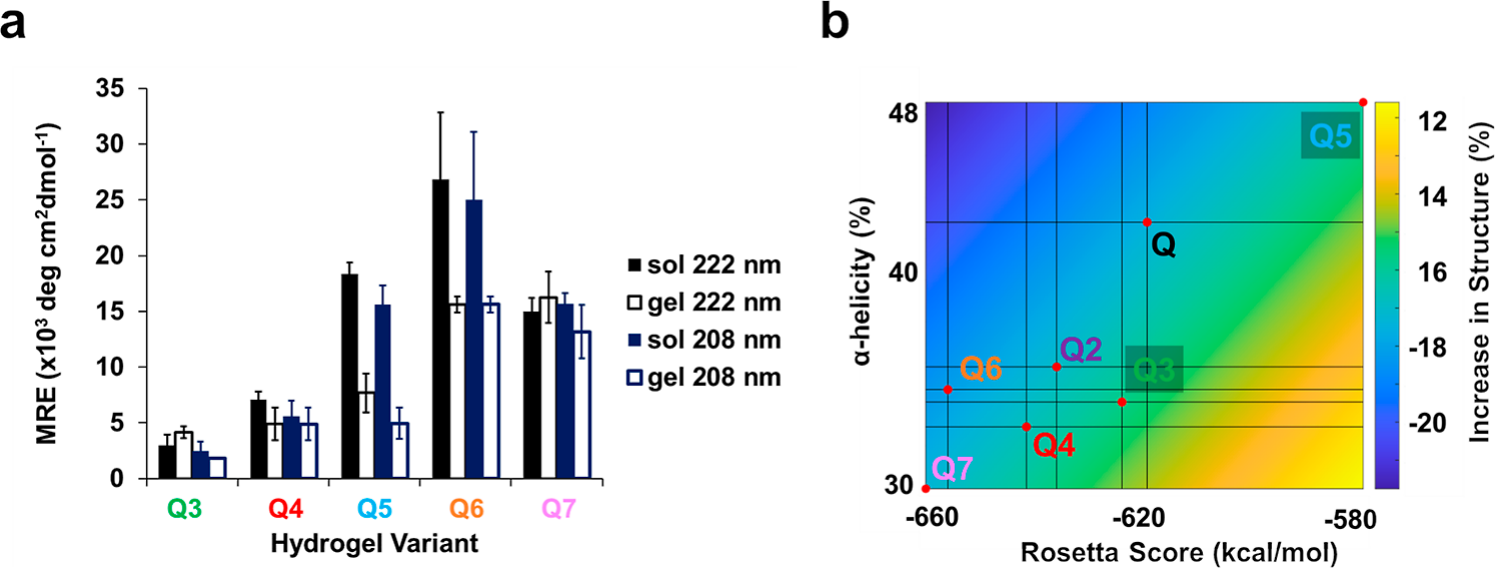
(a) Comparison of negative MRE values at helical minima 222 and 208 nm. Error bars represent the standard deviation from three independent trials. (b) Bivariate linear correlation between Rosetta score and hydrogel α-helicity and increase in structured content (α-helicity and β-sheet), as measured by deconvoluted ATR-FTIR spectra.

To assess the secondary structure of the hydrogels, circular dichroism (CD) spectroscopy was employed. New protein hydrogels Q3–7 were subjected to wavelength scans diluted in water to 15 μM, as done previously.^16^ CD spectra of all hydrogels exhibited double minima at 222 and 208 nm, indicative of helical secondary structure content (Table S3). Q3 and Q4 possessed reduced minima of −2500 ± 900 deg cm^2^ dmol^–1^ at 208 nm and −3000 ± 900 deg cm^2^ dmol^–1^ at 222 nm, and −5600 ± 1400 deg cm^2^ dmol^–1^ at 208 nm and 7100 ± 800 deg cm^2^ dmol^–1^ at 222 nm, respectively (Figure S12a,b), similar to the previously characterized hydrogels Q^15^ and Q2.^16^ However, Q5, Q6, and Q7 possessed significantly enhanced double minima (<10 × 10^3^ deg cm^2^ dmol^–1^), indicative of a more helical protein system (Figure S12c–e). Specifically, Q7 exhibited −15,700 ± 1000 deg cm^2^ dmol^–1^ at 208 nm and −15,000 ± 1200 deg cm^2^ dmol^–1^ at 222 nm, Q5 revealed −15,600 ± 1800 deg cm^2^ dmol^–1^ at 208 nm and −18,300 ± 1000 at 222 nm, and Q6 possessed −25,000 ± 6100 deg cm^2^ dmol^–1^ at 208 nm and −26,800 ± 6000 deg cm^2^ dmol^–1^ at 222 nm. Regardless, all protein hydrogels experienced a strong reduction in the helical signal (Figure 5a), consistent with our previous CD measurements of Q and Q2 after gelation. Conversely, Q hydrogel variants (with the exception of Q4) experience a significant shift toward a higher 222/208 ratio, indicative of increased α-helicity and overall coiled–coil structure.^42−44^

As gelation of the coiled–coil hydrogels is concentration- and buffer-dependent,^45^ ATR-FTIR spectroscopy was employed to deconvolute contributions by α-helical, β-sheet, and random coil content (Table S4). Previously, Q and Q2 protein hydrogels exhibited an increase in structured content (α-helical and β-sheet) after incubation at 4 °C and transformation from solution to gel by ATR-FTIR peak deconvolution. Interestingly, we observed a variety of overall changes in structured content in Q3-7 hydrogels in transition from solution (Figure S13a) to gel (Figure S13b). The increase in structured content is well-correlated with Rosetta energy scores (*R*^2^ = 0.78) with lower energy scores experiencing an improved change in structured content and higher energy scores experiencing a loss in structured content (Figure S13c). The Rosetta energy scores also possess a correlation with the α-helicity of the protein as a gel after incubation at 4 °C, where lower Rosetta energy scores positively correlate with lower α-helicity (Figure S13d). Naturally, this indicates a strong positive correlation with gel α-helicity and overall structural transition of the proteins from solution to gel (Figure S13e). Conversely, the α-helical content of the proteins after gelation holds a weak relationship to β-sheet content (*R*^2^ = 0.03) and to random coil content (*R*^2^ = 0.20), indicating the dependence of this transition on α-helicity.

The intercorrelation (*R*^2^ = 0.81) of structural transition, α-helicity, and Rosetta energy scores (Figure 5b) indicates that the Rosetta score is capable of a relative structural prediction of coiled–coil protein hydrogels where lower Rosetta energy scores indicate a lower helical content of the protein hydrogel and a loss of structured content and vice versa. Furthermore, Rosetta scores and secondary structure measurements are not strongly correlated to hydrogel strength, cross-linking, or critical gelation time. These relationships indicate the ability to use Rosetta scores to potentially predict structural transitions of coiled–coil protein hydrogels and their relative α-helical content as a hydrogel.

### Structural Dependence of Gelation Using Q5

**Figure 6:**
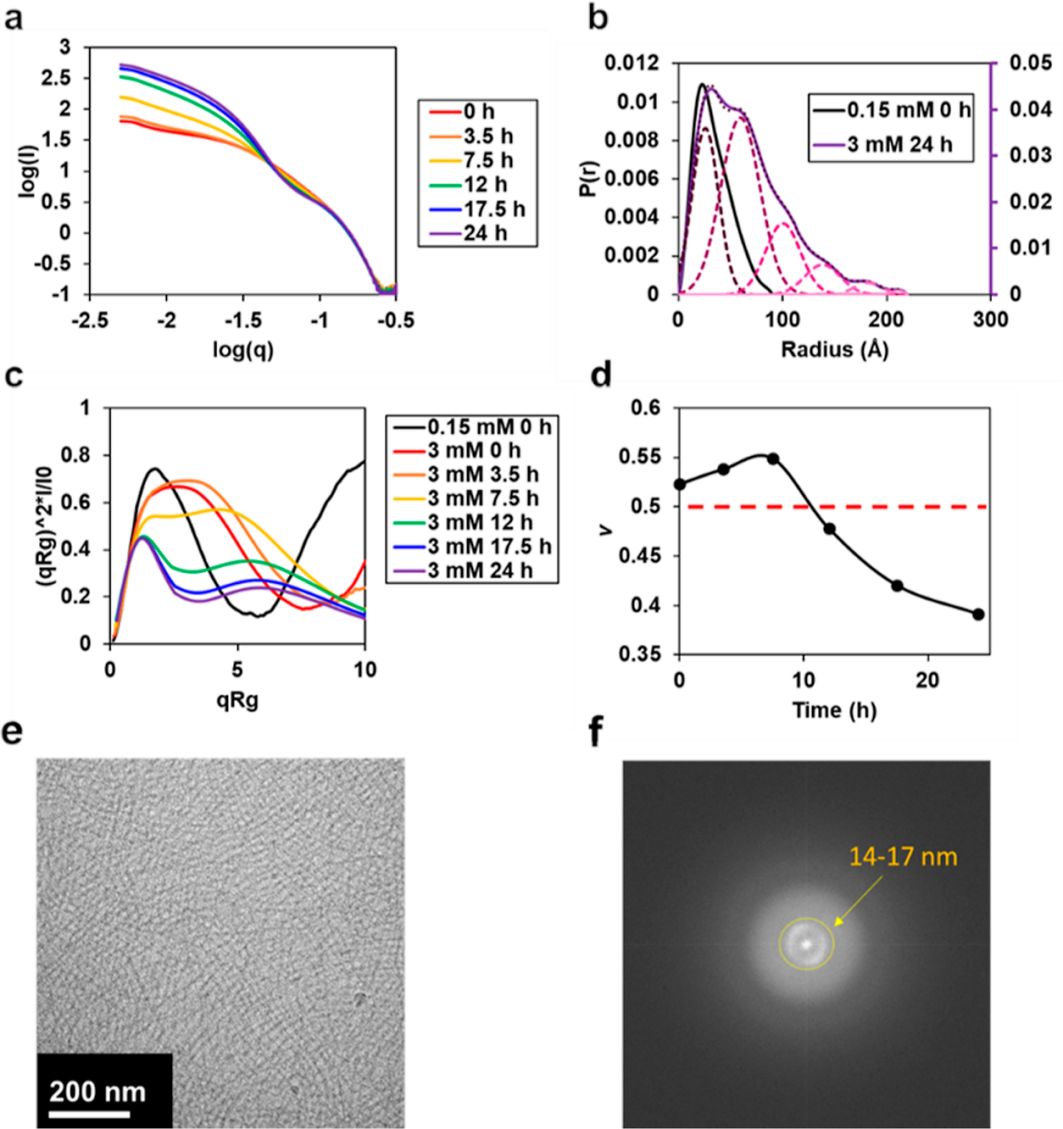
(a) Low *q* log–log SAXS spectra for Q5 at 3 mM and incubation at 4 °C from 0 to 24 h. (b) Pair distance distribution function calculated in primus (ATSAS software) for Q5 at 0.15 mM and 0 h of incubation at 4 °C (black line) and Q5 at 3 mM and 24 h of incubation at 4 °C (purple line) with Gaussian deconvolutions made in PeakFit software (magenta/pink dotted lines). (c) Kratky plots of Q5 at 0.15 mM and 0 h of incubation at 4 °C and Q5 at 3 mM and incubation at 4 °C from 0 to 24 h. (d) Flory exponent, ν, calculated using the MFF fit by the Sosnick group.^31^ (e) Representative cryo-EM image of Q5 at 2 mM and its (f) fast Fourier transfer (FFT) output.

Small angle X-ray light scattering (SAXS) is employed to determine nanoscale structural differences and the supramolecular assembly into nanofibers and physically cross-linked hydrogels. We exploit the fast gelation time of Q5 to explore the transition within a 24 h window using Life Science X-ray Scattering (LiX) beamline. At all measured time scales, Q5 exhibits an upward tail at the low q range characteristic of attractive interparticle interactions, expected for our self-assembling system (Figure 6a). Over time, a clear transition in scattering intensity also appears in the length scale between 10 and 32 Å, consistent with the interchain distance of a coiled–coil.^46^ This interparticle interaction is elucidated by indirect Fourier transform (IFT) into the pair distance distribution function, *P*(*r*), using primus (ATSAS software; Figure 6b).

To understand the distribution of coiled–coils independent of supramolecular assembly and higher order interparticle interaction, we have gathered a spectra near-representative of the form factor, *F*(*q*). The *P*(*r*) of Q5 at 150 μM concentration and 0 h of incubation at 4 °C (Figure 6b) indicates a monodisperse distribution centered at 22.5 Å, corresponding to the pore size of a coiled–coil.^47^ Upon gelation, new length scales of interparticle interactions are introduced as suggested by the *P*(*r*) of Q5 at 3 mM and 24 h of incubation at 4 °C. Peaks are deconvoluted using PeakFit software to *R*^2^ > 0.99, with deconvoluted peaks having full-width half-maximums (fwhm) at 25.5 59.2, 100.2, 138.3, 181.8, and 210.5 Å. Interestingly, these correspond to an average step-size increase of 39.1 ± 4.2 Å. Assuming a cylindrical shape from a coiled–coil, this size translates to a cylinder of length 6.8 ± 0.4 nm, which is consistent with the reported length of the parent coiled–coil sequence COMPcc of ∼7 nm,^47^ confirming a primarily longitudinal and length-wise assembly.

Using dimensionless Kratky analysis of SAXS measurements (Figure 6c), Q5 at relatively low molecular weights without incubation at 4 °C generates a well-folded domain indicated by the maximum at low *qR*_g_. However, the extended and divergent tails indicate that the protein system possesses flexibility. At high concentrations prior to gelation, Q5 is disordered and partially unfolded, as illustrated by the broad curve at 0 h. Over time, the extended plateau becomes diminished and the plot becomes compact, indicating a folded macromolecular structure.^48^ Furthermore, the divergence of the Kratky plot at high q and early times (0 and 3.5 h) indicates flexibility of the protein—a feature that becomes absent at high *q* and later times (≥7.5 h) demonstrated by the convergence to 0. The presence of two local maximums in the Kratky plot becomes apparent between 12 and 24 h, consistent with previous SAXS analysis of cross-linked thin fiber gel composed of low-molecular-weight organic gelators (LMOGs).^49^

Finally, we quantify the relative solvent–chain interaction of the Q5 protein using the polymer molecular form factor (MFF) fit, developed by the Sosnick group,^50^ to solve for the Flory exponent, ν (Figure 6d). Here, we observe that at low times, Q5 can be considered to possess relatively less favorable interchain interactions, where it exhibits values increasingly greater than 0.5 through 7.5 h, whereas at high times, Q5 can be considered to possess relatively more favorable interchain interactions, with values increasingly less than 0.5 from 12 to 24 h. The formation of a low-population of protofibrils facilitates high solvent–chain interaction indicated by ν > 0.5. Upon formation of a sufficient population of thin fibers, significant hydrophobicity is introduced into the system to cause nanofiber chain collapse and hydrophobicity as indicated by ν < 0.5. This is further confirmed by interval TEM measurements of Q5 over time at a high concentration (3 mM). At 3 h, a dark intensity in the background is seen as a result of unstructured protein aggregates with an ordered nanofiber assembly beginning (Figure S14a). At 6 h, nanofibers are formed together with a lower background intensity, representing the transition away from disordered aggregates (Figure S14b). At 12 h, a substantial number of long protein fibers form physical cross-links consistent with ν < 0.5 and high chain–chain interaction (Figure S14c). At 24 h and completion of gel formation, nanofibers appear uniformly physically cross-linked with a high contrast between the light background representative of low or no unordered protein aggregates and dark stained protein fibers (Figure S14d).

To support SAXS structural measurements, cryo-EM is employed to compare the preserved Q5 gel structure (Figure 6e). Cryo-EM images of Q5 at 2 mM reveal physically cross-linked diameters averaging 5.8 ± 1.5 nm (*n* = 100) (Figure S15a,b), significantly lower than as measured by TEM at 22.2 ± 8.4 nm. Moreover, at lower concentrations, Q5 nanofibers exhibit a distribution shift to smaller diameters where fiber diameters are 5.6 ± 2.1 nm (*n* = 100) at 1 mM (Figure S15c,d) and 3.4 ± 0.7 nm (*n* = 100) at 0.5 mM (Figure S15e,f), suggesting a concentration dependence for stable lateral fiber assembly. The higher fiber diameters measured in TEM may be explained by collapse and drying effects on the fibers, where the cryo-EM sizes are a better representation of the solution state diameters. Fast Fourier transfer (FFT) (Figure 6f) shows a concentric atomic spacing 14–17 nm from the center indicating that the fibers are spaced away in this range on average.

### Predicting Q5 Fibril Stability and Morphology from Molecular Dynamics

**Figure 7:**
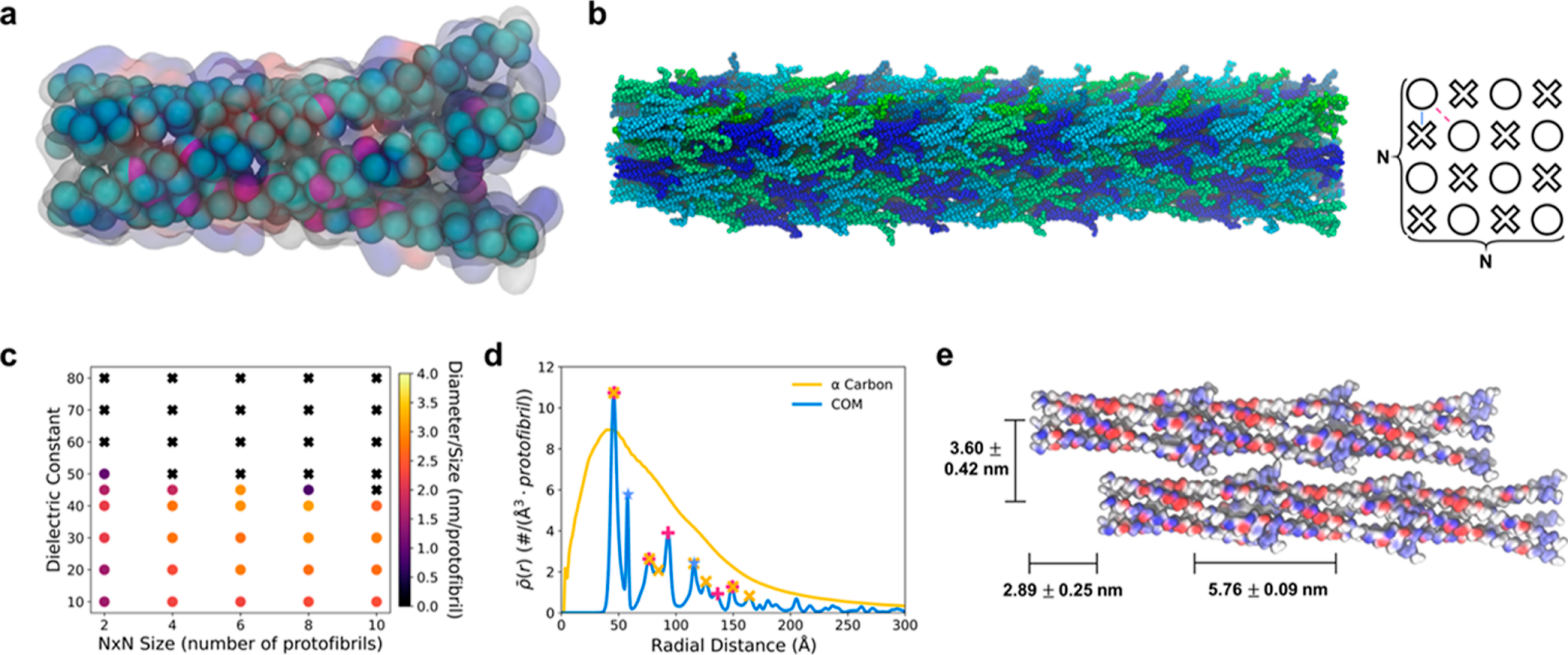
Coarse-grained (CG) molecular dynamics (MD) of Q5 fibrils. (a) Schematic of the Q5 coiled–coil (CC) CG model mapped to atomistic sites; the CG sites and virtual CG sites are represented as cyan and magenta spheres, respectively. (b) Snapshot from a CGMD simulation (ϵ = 40) of a 4 CC by 4 CC fibril consisting of protofibrils of length 14 CCs. Cyan, green, and blue colors are used to distinguish the individual CCs. The schematic shows the staggered square lattice configuration where “circles” indicate coplanar protofibrils and “crosses” indicate coplanar protofibrils that are staggered with respect to “circles”. The solid blue and dashed red lines indicate “adjacent” and “diagonally-aligned” protofibrils, respectively. (c) Phase diagram showing the average normalized fibril diameter predicted by CGMD simulations as a function of the dielectric constant and initial fibril size (*N* is the number of protofibrils used to construct the fibril based on an *N* × *N* grid). Here, circle (“○”) and cross (“×”) symbols represent fibrils that stay associated and become dissociated, respectively. (d) The normalized radial density distribution  between all CG sites (i.e., α carbon positions, yellow line) and between all centers-of-mass (COM, blue line) of each Q5 CC; the COM distribution is scaled by a factor of 20,000 to account for the relative difference in magnitudes of the two distributions due to differing total number densities. (e) Schematic showing the inter-CC alignment observed from CGMD simulations (ϵ = 40, 4 × 4 × 14 CCs); the surface representation is colored by charge and ranges between −0.75*e* (red) to 0.0*e* (white) to 0.75*e* (blue).

CG MD simulations were performed to investigate the importance of electrostatic interactions for physical cross-linking of Q5 protofibrils. The CG model of Q5 was mapped to and trained from atomistic MD statistics using hybrid bottom-up and top-down coarse-graining approaches (Figure 7a). CG Q5 coiled–coils were initially stacked into protofibrils, which were arranged into square lattices of varying *N* × *N* sizes with adjacent fibrils staggered along the fibril axis direction; an example 4 × 4 fibril after MD relaxation is shown in Figure 7b. Each fibril was simulated over a range of dielectric constants (ϵ) to assess the impact of changing the electrostatic interaction strengths on fibril stability (Figure S16). As a metric for stability, we computed the equilibrated diameter of each fibril normalized by the number of protofibrils (Figure 7c, Supporting Information in Figure S17). Fibrils that were stable adopted a mean diameter of 0.53 ± 0.09 nm/protofibril between 40 < ϵ < 50, while diameters decreased as ϵ decreased, with a mean diameter of 0.44 ± 0.01 nm/protofibril at ϵ = 10. Both phenomena indicate that a net attractive electrostatic interaction is present between protofibrils but is insufficient to promote fibrilization when ϵ > 50. In addition, we observed a positive correlation between the minimum electrostatic strength necessary for fibril stability and fibril size; only the 2 × 2 fibril was stable at ϵ = 50, while all fibril sizes were stable at ϵ = 40. Interestingly, the 4 × 4 and 6 × 6 fibrils, which yield diameters (7.33 ± 2.05 and 18.00 ± 5.10 nm, respectively) similar to that of our experiments, were stable at a maximum ϵ = 45, which is comparable to the average dielectric constant of water (ϵ = 80) and protein (ϵ ≈ 15).^51^ In our CG model, electrostatics are represented as point charges within a dielectric medium with screening due to mobile ions. Due to the low-resolution and implicit-solvent nature of our CG model, CG site interactions include both protein and water contributions to the dielectric medium. The screened nature of the electrostatics ensures that electrostatic interactions are only noticeable at small distances (<3 nm) with the contribution from the protein medium increasing as Q5 monomers approach each other. Hence, we interpret the effective ϵ = 45 as representative of the mixed protein/water dielectric environment that emerges during the short-range interactions between Q5.

Next, we computed normalized radial density distributions  for the 4 × 4 fibrils (Figure 7d, all other fibril sizes in Figure S18) to compare with SAXS distributions (Figure 6b). Both CGMD and SAXS distributions have peaks at distances of ∼40, ∼75, and ∼120 Å, suggesting that the predicted CGMD fibril morphologies are similar to those of experiments. We also computed  using only centers-of-mass of each coiled–coil to reveal the morphological features convolved within the original distribution (Figure 7d). Here, the blue stars, red plus symbols, and yellow crosses indicate peaks associated with stacked end-to-end coiled–coils (minimum distance of 5.8 nm), coiled–coils in adjacent protofibrils (blue line in Figure 7b) that are staggered (minimum distance of 4.6 nm), and coiled–coils in diagonally adjacent protofibrils (red line in Figure 7b) that are not staggered (minimum distance of 4.6 nm), respectively. The narrowness of the red-marked peaks indicates the stability of staggered protofibrils, likely due to charge complementarity, while the broadening of the yellow-marked peaks suggests that the stabilization afforded by charge complementary is local and does not fully compensate the electrostatic repulsion between nonstaggered, diagonally adjacent protofibrils. Finally, we note that while peak positions predicted by CGMD are analogous to that of SAXS experiments, the prominence of these peaks is not as distinct, which we attribute to the limited aspect ratio of our simulated fibrils, i.e., 14:4 (length/width) in the case of the 4 × 4 fibril.

Overall, our MD simulations suggest that fibrils are stabilized through electrostatic charge complementarity between adjacent protofibrils. This complementarity arose in the staggered square lattice structure present in Figure 7b, which we depict in Figure 7e. However, the complementarity-induced net attraction diminishes with size (as suggested by Figure 7c). This trend likely arises due to the increased ratio of diagonally adjacent, nonstaggered protofibrils to adjacent, staggered protofibrils as fibril width increases. Smaller fibrils, having a higher ratio of adjacent protofibrils to diagonal protofibrils, experience greater net electrostatic attraction compared with larger fibrils, having a smaller adjacent protofibril to diagonal protofibril ratio. As such, a maximum fibril diameter emerges, which our simulations predict to be around 19 nm (i.e., 6 × 6) in agreement with experimental Q5 diameters. Furthermore, our simulations were performed with the assumption of 0.5 M NaCl solvent, and we expect higher salt concentrations (i.e., greater electrostatic screening) to result in smaller fibrils. Nonetheless, we note that while our current study focused on the role of electrostatics for physical cross-linking of protofibrils, further work is necessary to decouple the possible contributions of nonelectrostatic interactions (e.g., dispersion forces and solvent-mediated forces). In addition, while we assumed a staggered square lattice structure that was both thermodynamically stable and consistent with experimental SAXS data, we cannot disregard other possible fibril morphologies. Additional work to investigate the distribution of morphologies that result from the self-assembly of Q5 and other Q variants is currently ongoing. Finally, we note that the computational modeling approach presented here, i.e., the complementary insights from Rosetta and CG modeling, can be applied to other Q variants or protein systems to assess the importance of electrostatically driven assembly.

## Conclusions

While coiled–coil chemical properties and oligomerization is a largely solved problem,^52^ their supramolecular assembly into fibers and hydrogels has not been well understood. Based on recent work showing a correlation between fiber assembly and the difference between electrostatic potential energy on the surface of opposing termini,^17^ we develop a model to predict and design coiled–coil hydrogels with fiber diameters and gelation rates. We also establish important correlations among coiled–coil protein structure, thermostability, and Rosetta energy scores. Furthermore, we show that protein structure dictates the ability for coiled–coil protein hydrogels to possess blue autofluorescence, which establishes the coiled–coil proteins designed here to be the first known hydrogels with the capability. Finally, using a combination of SAXS and MD simulations, we show a molecular understanding of coiled–coils that self-assemble into protofibrils and then fibrils as well as their relative probabilistic geometries, which we demonstrate is due to charge complementarity at both end-to-end and staggered side-to-side coiled–coil interfaces. Overall, our investigation allows for the first top-to-bottom understanding and bottom-up design of the coiled–coil supramolecular assembly and in turn generates models and tools for their predictive design. This provides the basis to systematically design and tune coiled–coil hydrogels and a foundation for understanding other electrostatically driven molecular interactions.

## Data Availability

Code availability: https://github.com/drb450/CoiledCoilDesign.

## Supporting Information Available

Q3–7 protein purification; preliminary regression for ΔEE_bcf_ and *t*_c_ using variants Q, Q2, Q4, and Q5; Q3–7 UCST phase diagrams; bivariate linear regression heatmap and accuracy; average and standard deviation of starting fluorescence prior to FRAP experiments; Q3–7 CD spectra with 222/208 nm comparison; Q3-Q7 ATR-FTIR spectra in solution and gel state and intercorrelation between helicity and Rosetta score; time-course Q5 TEM images; concentration-dependent cryo-EM images of Q5; energy surface plots for Q5MD simulations; radial density distributions of Q5MD simulations; normalized number density plots of fibrils in Q5MD simulations; average and standard deviation values for Q3–7 222 and 208 nm MRE values measured by CD; and ATR-FTIR percent deconvolution for the secondary structure of Q3–7 protein as a solution and hydrogel after incubation at 4 °C (PDF)
