# Correction to “Computational Prediction of Coiled–Coil Protein Gelation Dynamics and Structure”

**DOI:** 10.1021/acs.biomac.4c00448

**Published:** 2024-04-17

**Authors:** Dustin Britton, Luc F. Christians, Chengliang Liu, Jakub Legocki, Yingxin Xiao, Michael Meleties, Lin Yang, Michael Cammer, Sihan Jia, Zihan Zhang, Farbod Mahmoudinobar, Zuzanna Kowalski, P. Douglas Renfrew, Richard Bonneau, Darrin J. Pochan, Alexander J. Pak, Jin Kim Montclare

The following Acknowledgment is being added to the published version.

