# Supplementary material for: Computational Prediction of Coiled–Coil Protein Gelation Dynamics and Structure
Source: Biomacromolecules. 2023 Dec 18;25(1):258–71. doi: 10.1021/acs.biomac.3c00968 (PMC10777397; doi:10.1021/acs.biomac.3c00968)
Supplement: Supplementary file 1 — bm3c00968_si_001.pdf [file bm3c00968_si_001.pdf]

**Supporting Information**

# Computational prediction of coiled-coil protein gelation dynamics and structure

*Dustin Britton[†], Luc F. Christians[⊿], Chengliang Liu[†,] Jakub Legocki[†], Yingxin Xiao[†], Michael Meleties[†], Lin Yang[♀], Michael Cammer[¥,] Sihan Jia[†,] Zihan Zhang[§], Farbod Mahmoudinobar[†,‖], Zuzanna Kowalski[†], P. Douglas Renfrew[‖], Richard Bonneau[‖,⊥,#], Darrin Pochan[§], Alexander J. Pak[⊿,∴], and Jin Kim Montclare[†,¶,∇,♀,£,*]*

[†] Department of Chemical and Biomolecular Engineering, New York University Tandon School of Engineering, Brooklyn, NY, 11201, USA

[⊿] Department of Chemical and Biological Engineering, Colorado School of Mines, Golden, CO, 80401, USA

[♀] National Synchrotron Light Source – II, Brookhaven National Laboratory, Upton, NY 11973, USA

[¥] Microscopy Laboratory, New York University Langone Health, New York, NY, 10016, USA

[§] Department of Materials Science and Engineering, University of Delaware, Newark, DE, 19716, USA

[‖]. Center for Computational Biology, Flatiron Institute, Simons Foundation, New York, New York, 10010, USA

[⊥]. Center for Genomics and Systems Biology, New York University, New York, New York, 10003, USA

[#]. Courant Institute of Mathematical Sciences, Computer Science Department, New York University, New York, New York, 10009, USA

∴ Quantitative Biosciences and Engineering, Colorado School of Mines, Golden, CO, 80401, USA

[∇] Department of Chemistry, New York University, New York, NY, 10012, USA

£ Department of Biomedical Engineering, New York University, New York, NY, 11201, USA

¶.Bernard and Irene Schwartz Center for Biomedical Imaging, Department of Radiology, New York University School of Medicine, New York, NY, 10016, USA

° Department of Biomaterials, New York University College of Dentistry, New York, NY, 10010, USA

* Corresponding authors

Email: montclare@nyu.edu

## Methods

**Protein Expression.** Q variants were expressed as described previously[1]. Plasmids encoding the Q variants were designed in pQE60 vectors and purchased from Genscript. Colonies were selected from tryptic soy agar plates possessing chemically transformed M15MA *E. coli* cells. Protein expression was induced in supplemented M9 media, started in 16 mL of starter culture, using 200 μg/mL IPTG when the optical density at 600 nm ($OD_{600}$) grew to 0.8-1.0. The culture was incubated at 37˚C and 350 rpm with an Innova 42 incubator shaker (New Brunswick), allowing the protein to express for 3 h post-induction. Cells were centrifuged at 5,000 × g at 4˚C for 30 minutes in an Avanti J-25 centrifuge (Beckman Coulter) and stored at -20˚C until purification. 12% SDS-PAGE was used to confirm expression.

**Protein Purification.** Q variants were purified as described previously[1]. Pelleted cells were first resuspended in 40 mL of Buffer A (50 mM Tris-HCl, 500 mM NaCl, pH 8.0) and lysed using a Q500 probe sonicator (QSonica) at 55% amplitude for 2 minutes with 5 s and 5 s off. Lysed cells were pelleted at 11,000 × g for 50 minutes to remove cell debris. Lysate was flowed through a cobalt-charged HiTrap IMAC FF 5 mL column and eluted at increasing concentrations of Buffer B (50 mM Tris-HCl, 500 mM NaCl, 500 mM imidazole). Fractions were assessed for purity by 12% SDS-PAGE and pure fractions were dialyzed using 6 consecutive buckets of Buffer A at 5 L volumes in 3.5 kDa MWCO snakeskin tubing. Dialyzed pure protein was then concentrated using 3 kDa MWCO Macrosep and Microsep Advance centrifugal devices (Pall Corporation) to 2 mM within six hours of removal from the dialysis bags. Protein concentration was determined by bicinchoninic acid (BCA) assay with a standard curve made using dilutions of bovine serum albumin (BSA).

**Microrheology.** Microrheology was used to assess gelation kinetics as described previously[2]. Immediately after concentration to 2 mM, 29.7 uL were aliquoted with 0.3 uL of 1 um diameter FluoSpheres and loaded into glass capillary tubes (VitroCom). Samples were then imaged using an inverted fluorescent microscope (ZEISS Microscopy) at 40X magnification with 2×2 binning. Samples were incubated at 4˚C on a rotisserie at 8 rpm between measurements and imaged incrementally at select time points. Relaxation exponents were tracked until a negligible difference was observed with the relaxation exponent of the previous timepoint. Images were stacked, converted to greyscale, and analyzed with multiple particle tracking (MPT) in MATLAB (Mathworks, R2021a) using code developed in-house and originally developed and modified by Dufresne, Kilfoil, Blair, and O'Neill as done previously[3]. Prior to other experiments of hydrogels in the gel state, microrheology here was used to confirm its complete transition.

**Rheology.** Q hydrogel variants were assessed for relative mechanical integrity with a stress-controlled rheometer (Discovery Hybrid Rheometer 2, TA Instruments) equipped with a parallel plate geometry. In parallel with microrheology measurements, 100 µL of each variant was incubated at 4˚C. Upon gelation as assessed by microrheology, hydrogels were loaded onto an 8 mm diameter lower and upper plate with a 0.2 mm geometry gap. Storage modulus (G') and loss modulus (G'') were measured from 0.1 – 10 Hz with 5% oscillation strain[1].

**Circular Dichroism Spectroscopy.** Secondary structure of protein samples pre- and post-gelation was assessed at 15 µM and 4˚C using a Jasco J-815 CD spectrometer with a PTC-423S single position Peltier temperature control system. The secondary structure of protein in solution was measured immediately after concentration to 2 mM, with protein diluted down to 15 µm using water to minimize salt interference. Secondary structure of protein in the gel state was measured under the same conditions after confirming gelation via microrheology. Wavelength scans were

performed from 195 to 250 nm at 1 nm step sizes and mean residue ellipticity (MRE) was calculated as described previously[4].

**Attenuated Total Reflectance-Fourier Transform Infrared Spectroscopy.** Secondary structure before (solution) and after gelation (gel) was also assessed by peak deconvolution of attenuated total reflectance-Fourier transform infrared (ATR-FTIR) spectra. ATR-FTIR measurements were performed using a Nicolet 6700 Fourier Transform Infrared Spectrometer equipped with a mercury cadmium telluride (MCT)-A detector. Spectra were collected for 5 μL samples of protein at 2 mM from 4000-400 $cm^{-1}$ with a 4.0 $cm^{-1}$ resolution, normalized, and buffer-subtracted prior to analysis from 1700-1600 $cm^{-1}$, corresponding to the amide I region[5]. ATR-FTIR measurements were performed immediately after concentration to 2 mM for solution measurements and after. Peaks were deconvoluted using Gaussian functions in PeakFit software until the goodness of fit reached $r^2 \geq 0.99$[6, 7].

**Transmission Electron Microscopy. T**ransmission electron microscopy (TEM) images were taken with a FEI Talos L120C transmission electron microscope. Samples were diluted to 50 μM and 3 μL was spotted on Formvar/carbon-coated copper grids followed by a 5 μL wash with water, and 3 μL staining with 1% v/v uranyl acetate solution each with incubation times of 1 min. Between steps, filter paper was used to gently wick the grids dry. Following imaging, minimum diameter nanofibers within the physically crosslinked hydrogel were sized in ImageJ software (Version 1.52q)[8].

**Small Angle X-ray Scattering (SAXS).** All measurements were taken within 12 h of concentration of the protein from the dialysis bag. To study Q5 as an isolated particle, 150 μM (~1 mg/mL) protein and dialysis buffer (50 mM Tris 500 mM NaCl pH 8.0) was loaded into a flow

cell and measured in the q range 0.005 $Å^{-1}$ to 3.19 $Å^{-1}$. The Q5 protein transition from solution to hydrogel was measured by incubation at 4˚C of 3 mM of Q5 protein in an eight-slot holder capable of loading 30 μL of each sample. Between measurements, the holder was returned for incubation at 4˚C. Independent slots were used to load six samples of the Q5 hydrogel in solution including one for the empty cell and one for the dialysis buffer. Each slot was measured once incrementally over a 24 h incubation window to negate the impact of radiation (except for the measurement made at 24 h which was measured from the previous time point sample due to sample error in the last slot holder, however, a negligible impact of radiation was observed). Similarly, Q5 hydrogel measurements made at 3 mM and 4˚C were measured in the q range 0.005 $Å^{-1}$ to 3.19 $Å^{-1}$. Kratky plots were calculated by Guinier analysis of Rg and I(0) values. Flory exponents were calculated using MFF fit by Sosnick group online tool[9, 10]. Pair distance distribution function, P(r), was calculated using primus (ATSAS software).

**Cryo-EM**. Cryo-EM samples were prepared with the Vitrobot system (Thermo Fisher Scientific) which can help vitrify a thin solution layer at low temperature. Approximately 4 μl of sample solution was applied to the grids in the chamber of Vitrobot at 4℃ and 100% humidity. The sample solution was incubated for 10 s and then the grid was blotted three times. Blotting lasted 1 s for each time. The grid was plunged into the liquid ethane (around -175°C) quickly to get the extremely fast cooling rate and the thin homogeneous vitreous layer. Then, the grid was transferred into liquid nitrogen. The cryo-EM holder was kept at −175°C to prevent solvent crystallization during the imaging.

**Coarse-Grained Model Details.** Each bond is defined by **Equation S1**:

$$U_{bonded}(r_{ij}) = K_{ij}(r_{ij} - r_{ij,0})^2 \qquad \textbf{Equation S1}$$

where $K_{ij}$ is the spring constant in kcal/mol/Å$^2$, $r_{ij,0}$ is the mean bond distance, and $r_{ij}$ is the pair distance between sites $i$ and $j$. Parameters were determined by training an Heteroelastic network model (HENM) using the CG-mapped atomistic data with added virtual sites as reference. Monomers were isolated and trained as separate frames within the dataset. Electrostatic potentials were defined using the Yukawa potential (**Equation S2**):

$$U_{coul}(r_{ij}) = \frac{Cq_iq_j}{\epsilon r_{ij}} e^{-\kappa r_{ij}} \qquad \textbf{Equation S2}$$

where $C$ is a unit conversion constant, $q$ is the charge in $e$, $\varepsilon$ is the dielectric constant, which was varied between 10 and 80, and $\kappa$ is the inverse Debye length, which was set to 0.234 Å$^{-1}$ based on 0.5 M NaCl at 4°C. Excluded volume potentials were defined based on soft exclusions (**Equation S3**):

$$U_{excluded}(r_{ij}) = D\left[1 + \cos\left(\frac{\pi r_{ij}}{r_c}\right)\right] \qquad r_{ij} < r_c \qquad \textbf{Equation S3}$$

where $D$ was set to 20.0 kcal/mol, which was set to be large enough to prevent CG site overlap while not being so large as to introduce numerical instability, and $r_c$ was set to 3.0 Å based on the minimum intermolecular CG site separation distance observed from the CG-mapped atomistic

trajectories. The virtual site potentials were defined between the virtual site and the real site it was mapped onto in the form of a Gaussian potential (**Equation S4**):

$$U_{vs}(r_{ij}) = -Ae^{-Br_{ij}^2} \quad \textbf{Equation S4}$$

where $A$ was set to 10.0 kcal/mol and $B$ was set to 0.2 Å$^{-2}$ to ensure enough attractive strength to maintain the coiled-coil and protofibril structures. These parameters were determined by running coiled-coil forming and protofibril forming CG MD simulations over a range of $A$ and $B$ values until the target structures were observed to form and remain stable. As seen in **Figure S20**, the explicit value of $A$ does not seem to influence the stability of fibrils across $\epsilon$ as long as $A$ is large enough to maintain protofibrils; $A$ = 6.2 kcal/mol is the minimum value of $A$ required to maintain protofibrils. Finally, we note that this model can be considered an implicit-solvent model as only protein is mapped while using atomistic statistics from solvated protein trajectories.

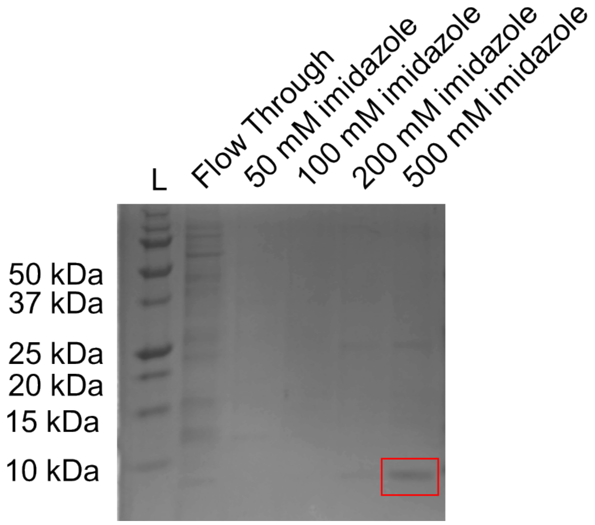


**Figure S1** Q3 protein after purification. L: ladder, following are increasing mM concentrations of imidazole.

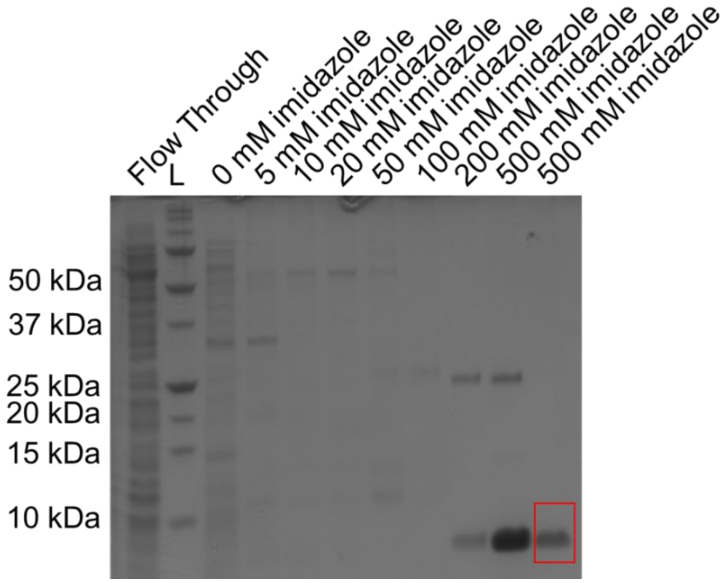


**Figure S2** Q4 protein after purification. L: ladder, following are increasing mM concentrations of imidazole.

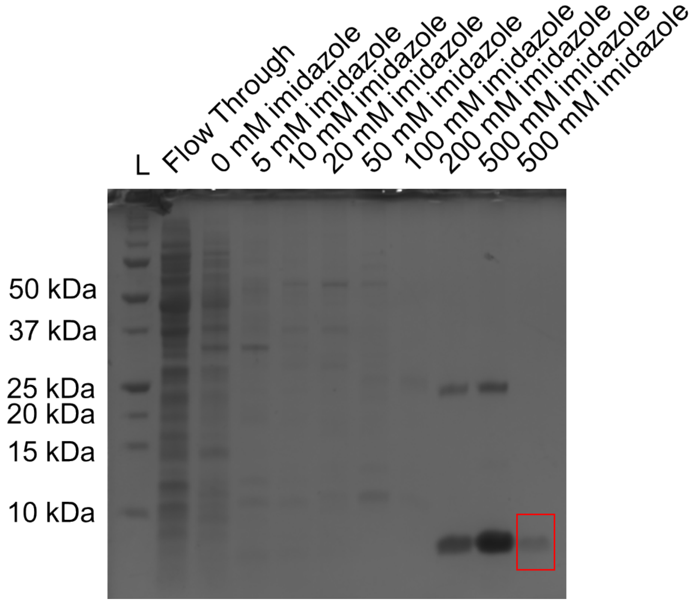


**Figure S3** Q5 protein after purification. L: ladder, following are increasing mM concentrations of imidazole.

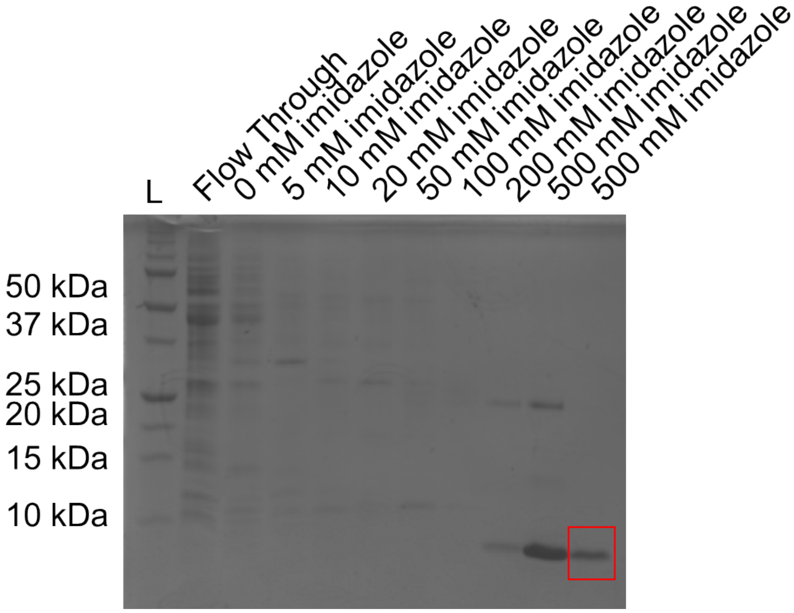


**Figure S4** Q6 protein after purification. L: ladder, following are increasing mM concentrations of imidazole.

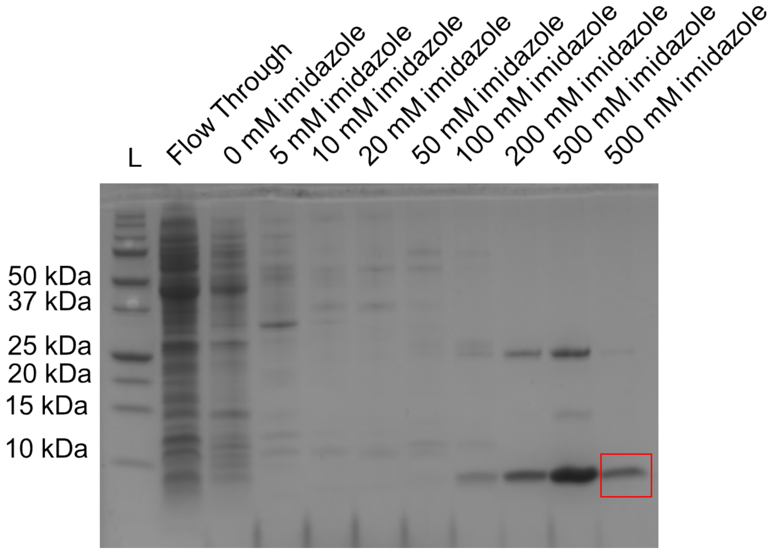


**Figure S5** Q7 protein after purification. L: ladder, following are increasing mM concentrations of imidazole.

| | Q | Q2 | Q3 | Q4 | Q5 | Q6 | Q7 |
|---|---|---|---|---|---|---|---|
| **Q** | 1.0000 | 0.0001 | 0.0050 | 0.0001 | 0.0001 | 0.8434 | 0.1271 |
| **Q2** | 0.0001 | 1.0000 | 0.0028 | 0.1662 | 0.0001 | 0.0001 | 0.0001 |
| **Q3** | 0.0050 | 0.0028 | 1.0000 | 0.0001 | 0.0001 | 0.0003 | 0.0180 |
| **Q4** | 0.0001 | 0.1662 | 0.0001 | 1.0000 | 0.0001 | 0.0001 | 0.0001 |
| **Q5** | 0.0001 | 0.0001 | 0.0001 | 0.0001 | 1.0000 | 0.0001 | 0.0001 |
| **Q6** | 0.8434 | 0.0001 | 0.0003 | 0.0001 | 0.0001 | 1.0000 | 0.0742 |
| **Q7** | 0.1271 | 0.0001 | 0.0180 | 0.0001 | 0.0001 | 0.0742 | 1.0000 |

**Figure S6** P-value chart comparing fiber diameter populations measured by TEM for Q,Q2-7 with red shading noting values not considered statistically significant by conventional criteria and green shading noting values considered statistically significant by conventional criteria.

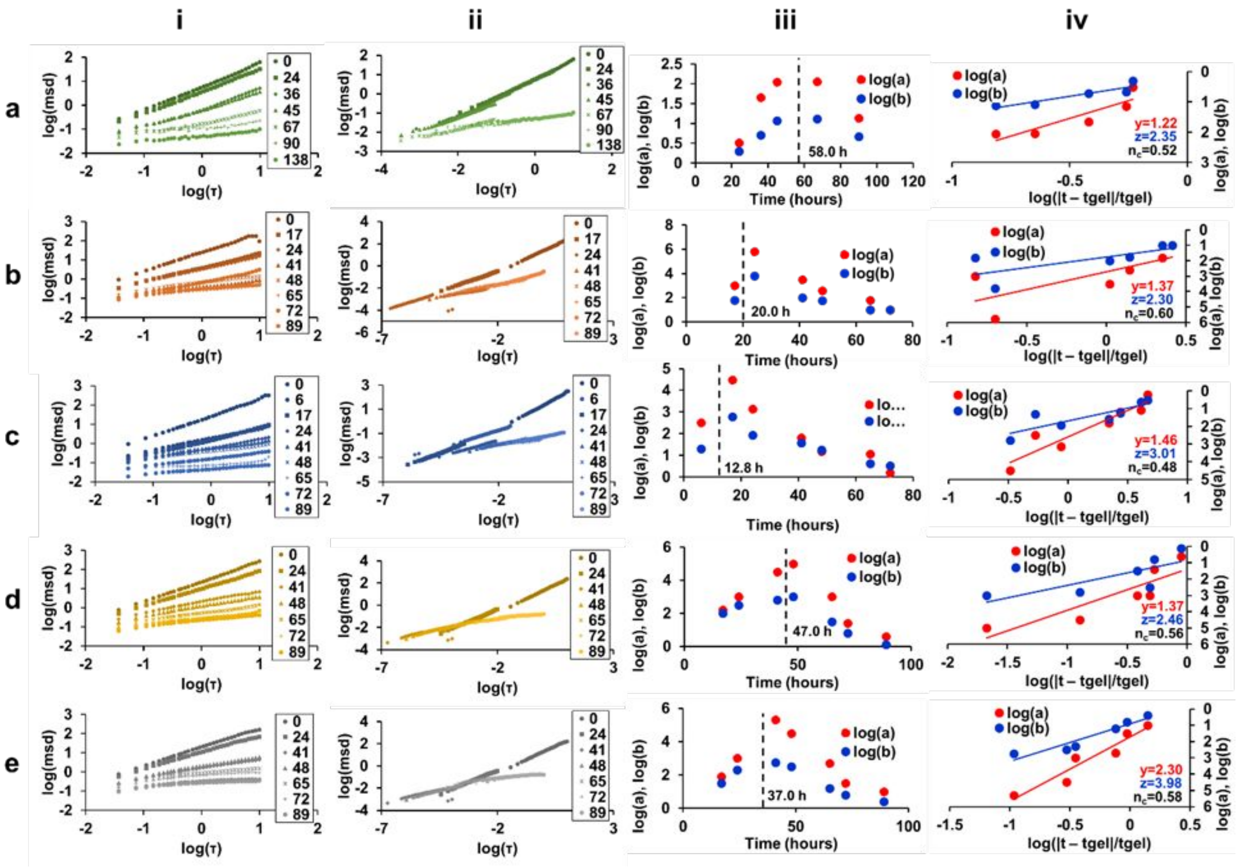

**Figure S7** Representative microrheological analysis using MPT for **a.** Q3, **b.** Q4, **c.** Q5, **d.** Q6 and **e.** Q7 showing **i** log-log plot of MSD and lag time, τ, **ii**. time-cure superposition of MSD vs τ, **iii**. Logarithmic shift factors for the vertical (log(a) in blue) and horizontal (log(b) in red) directions used in the time cure superposition to determine the $t_c$ and **iv**. log-log plot of the shift factors and their distance from $t_c$ determined by the ratio of the logarithmic slopes of the horizontal to vertical shift factor.

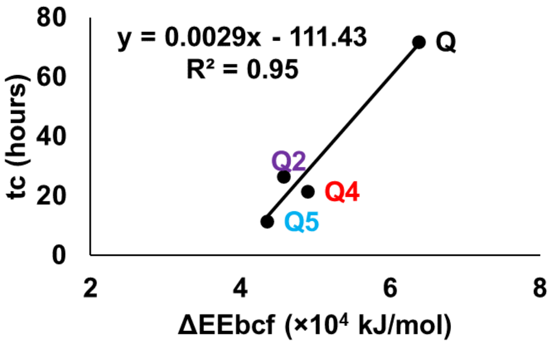


**Figure S8** Preliminary linear correlation for $\Delta EE_{bcf}$ to $t_c$.

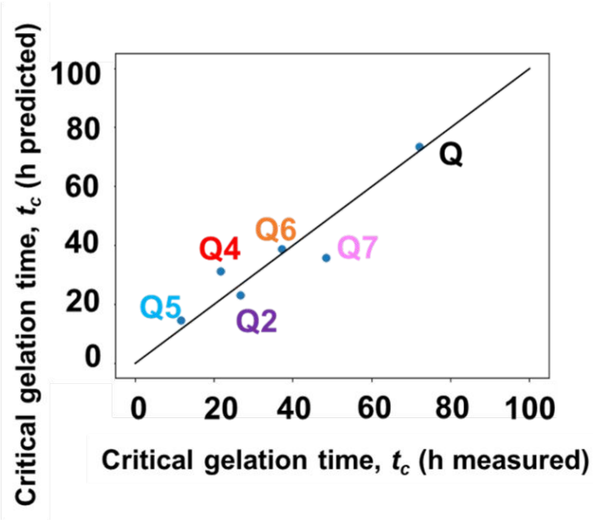


**Figure S9.** Accuracy of bivariate regression model for $NEE_{bcf}$ and $CEE_{bcf}$ to predict $t_c$ showing residual differences between measured $t_c$ and predicted $t_c$ using the bivariate linear regression model.

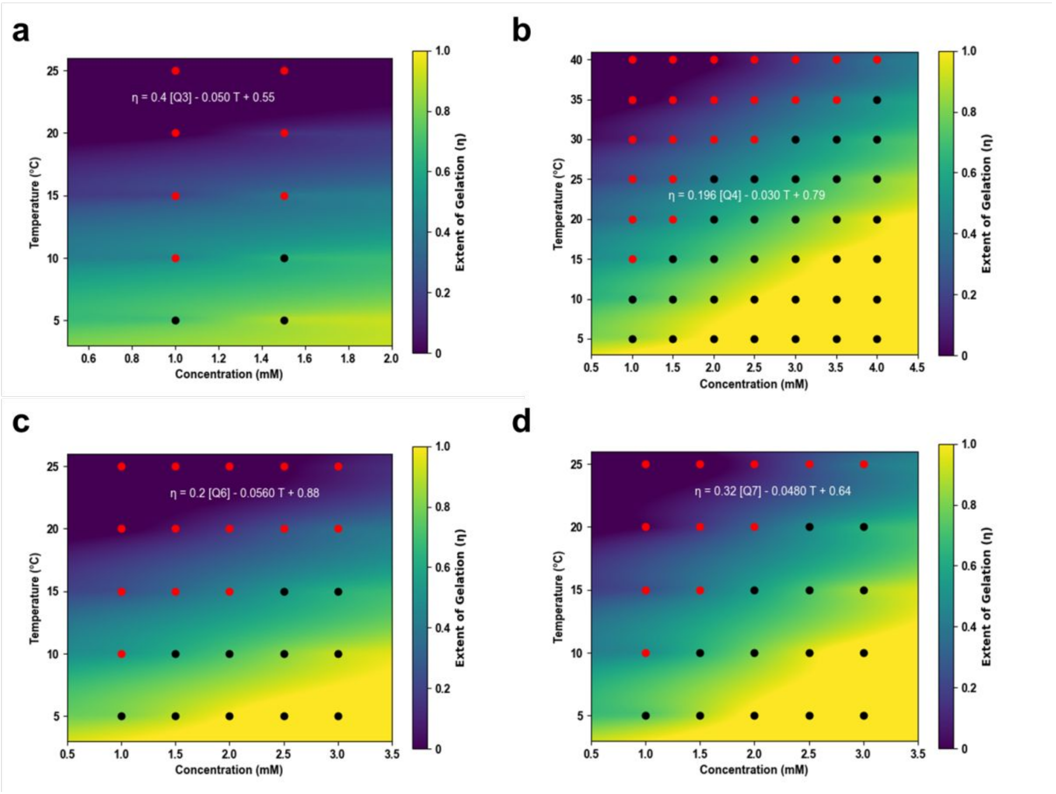


**Figure S10** Extent of gelation, η, calculated by bivariate linear regression of a concentration-temperature phase diagram constructed from tube inversions where black dots represent gel behavior and red dots represent solution behavior after two weeks of incubation at 4 °C for **a.** Q3, **b**. Q4, **c.** Q6 **d.** and Q7.

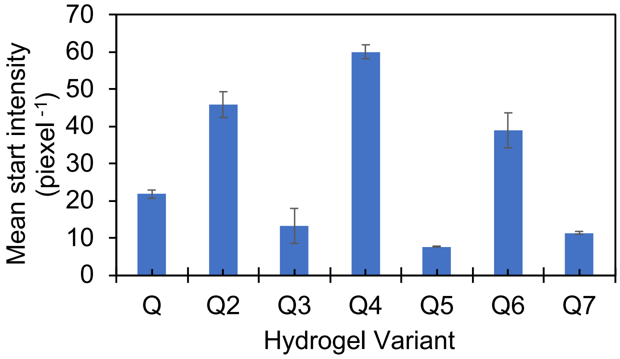


**Figure S11** Start intensity of hydrogel variants prior to FRAP experiment. Error bars represent standard deviation of three independent trials.

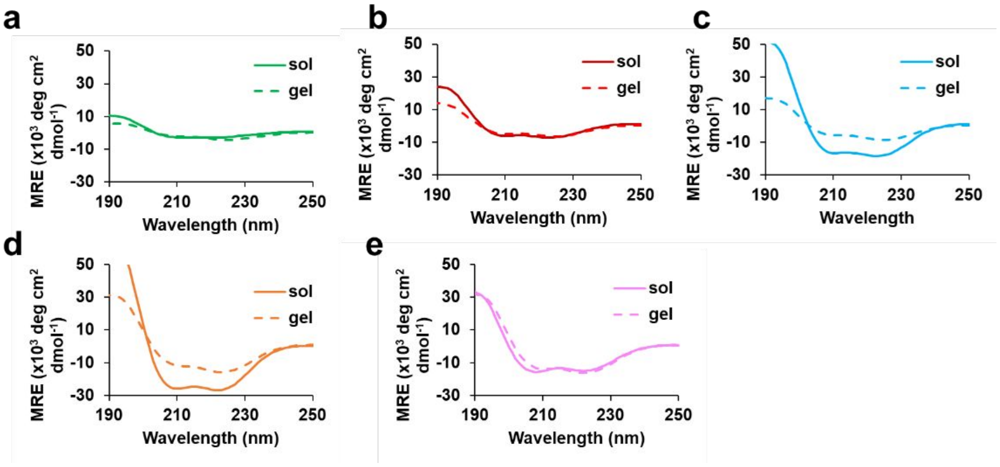


**Figure S12** Average CD spectra for **a**. Q3, **b.** Q4, **c**. Q5, **d**. Q6, and **e**. Q7 in solution state (prior to incubation at 4 °C, solid lines) and as a hydrogel (after incubation at 4 °C, dotted lines).

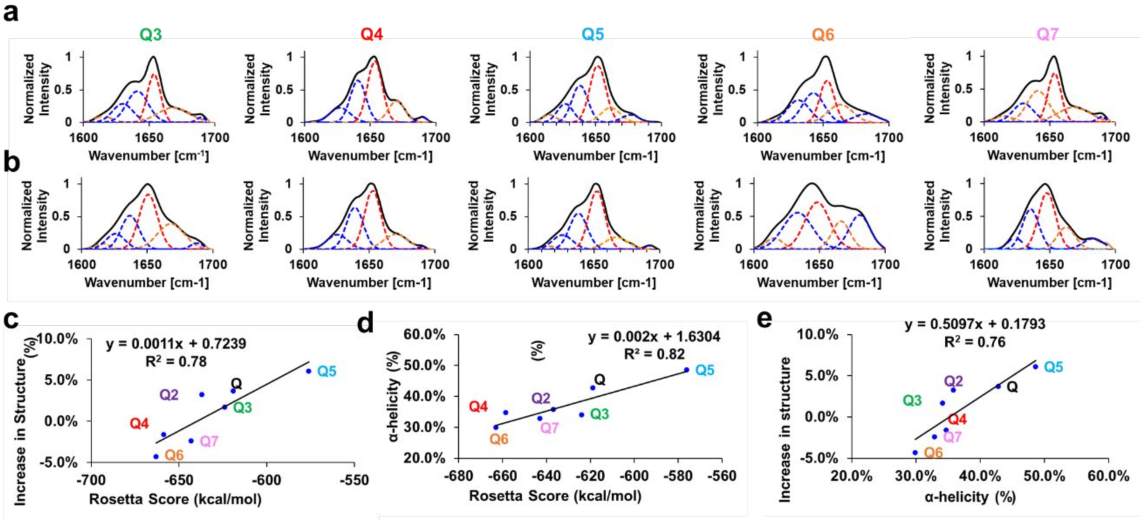


**Figure S13 a**. Representative ATR-FTIR spectra for Q3-7 in a solution state (pre-incubation at 4˚C) and **b.** as a hydrogel (post-incubation at 4˚C). Linear correlation of Rosetta score to **c**. percent increase in structured content (α-helix and β-sheet) between solution state and hydrogel ATR-FTIR spectra and **d**. deconvoluted α-helicity of ATR-FTIR spectra as a hydrogel. **e**. Linear correlation of α-helicity of ATR-FTIR spectra as a hydrogel and percent increase in structured content (α-helix and β-sheet) between solution state and hydrogel ATR-FTIR spectra.

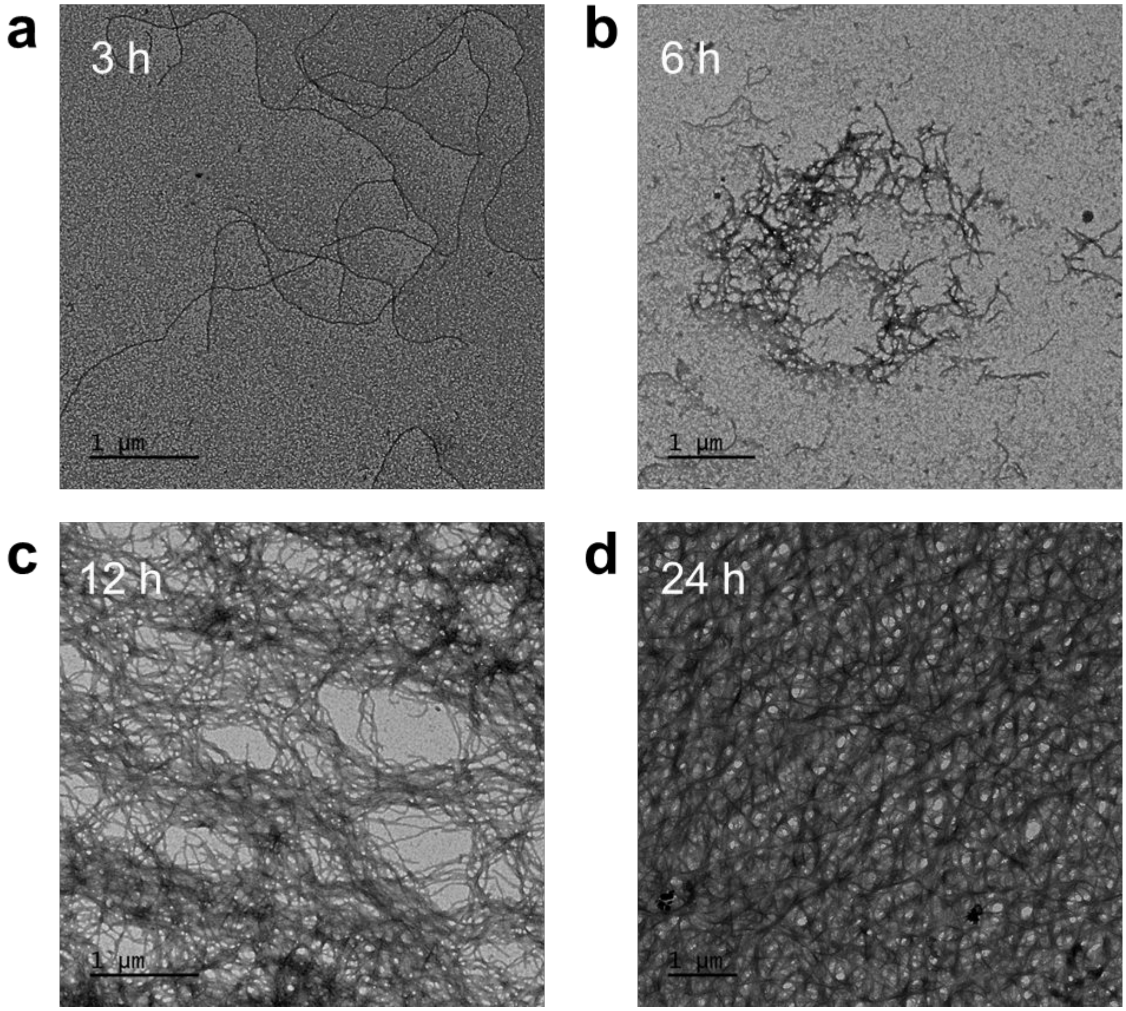


**Figure S14** Representative TEM images of Q5 after incubation at **a.** 3 h **b.** 6 h **c.** 12 h and **d.** 24 h incubated at 4 °C

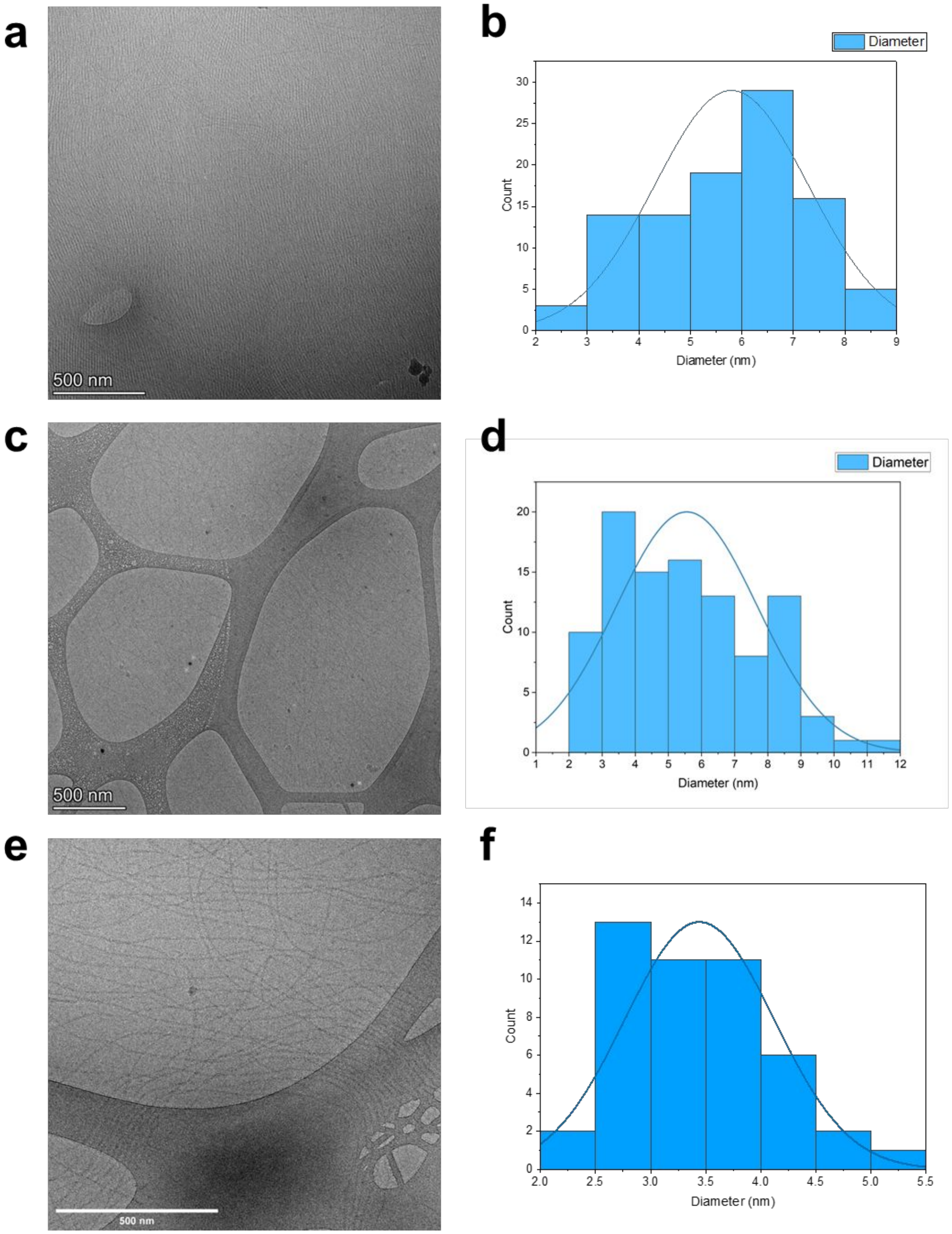


**Figure S15 a**. Representative cryo-EM images and **b**. corresponding histogram of measured fiber diameters of Q5 at 2 mM **c**. Representative cryo-EM images and **d**. corresponding histogram of measured fiber diameters of Q5 at 1 mM. **e**. Representative cryo-EM images and f. corresponding histogram of measured fiber diameters of Q5 at 0.5 mM

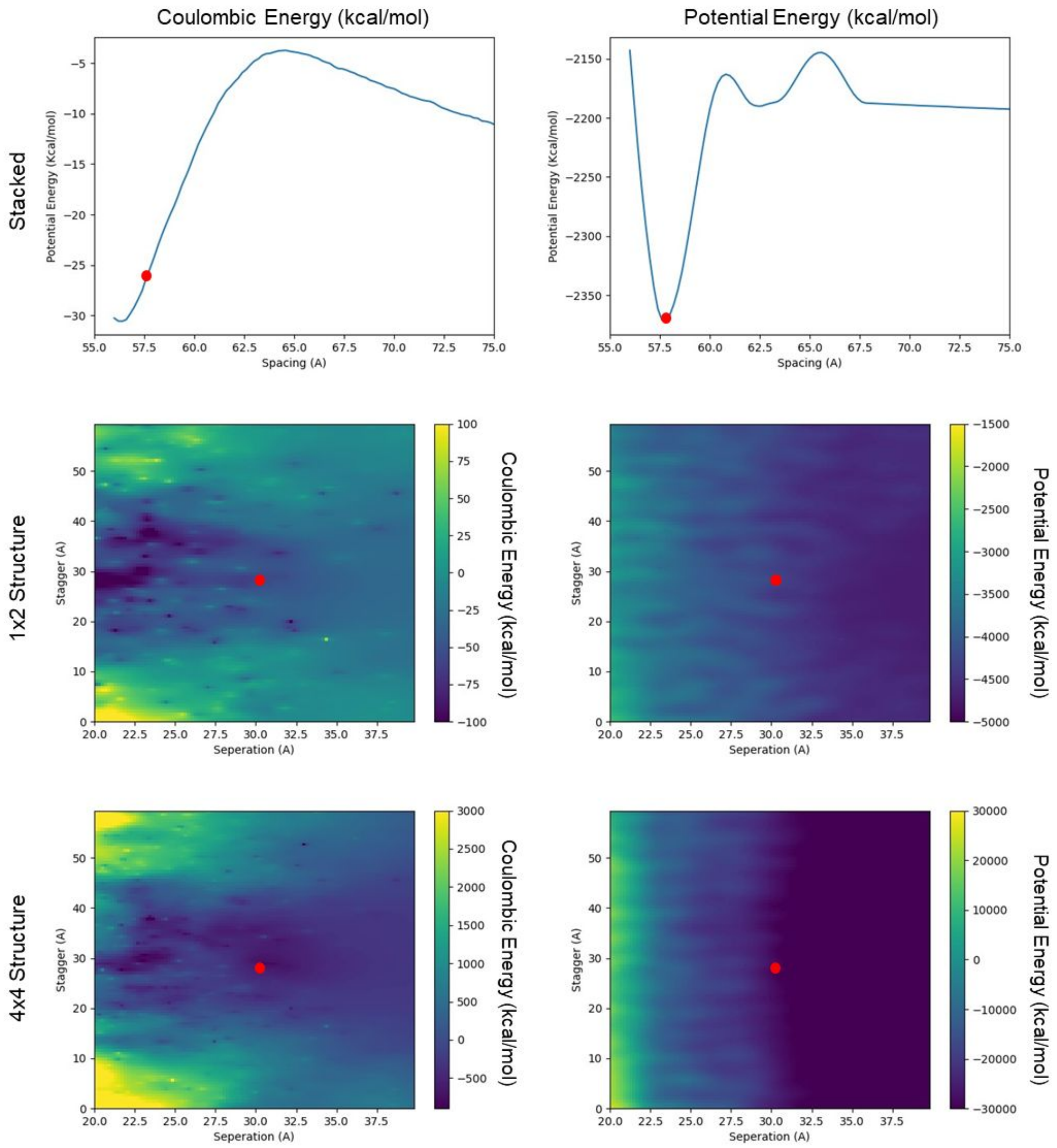


**Figure S16**. Energy surfaces for the CG model of Q5 calculated using LAMMPS. The red points indicate the initial separation and stagger values used to prepare the CG MD fibril simulations.

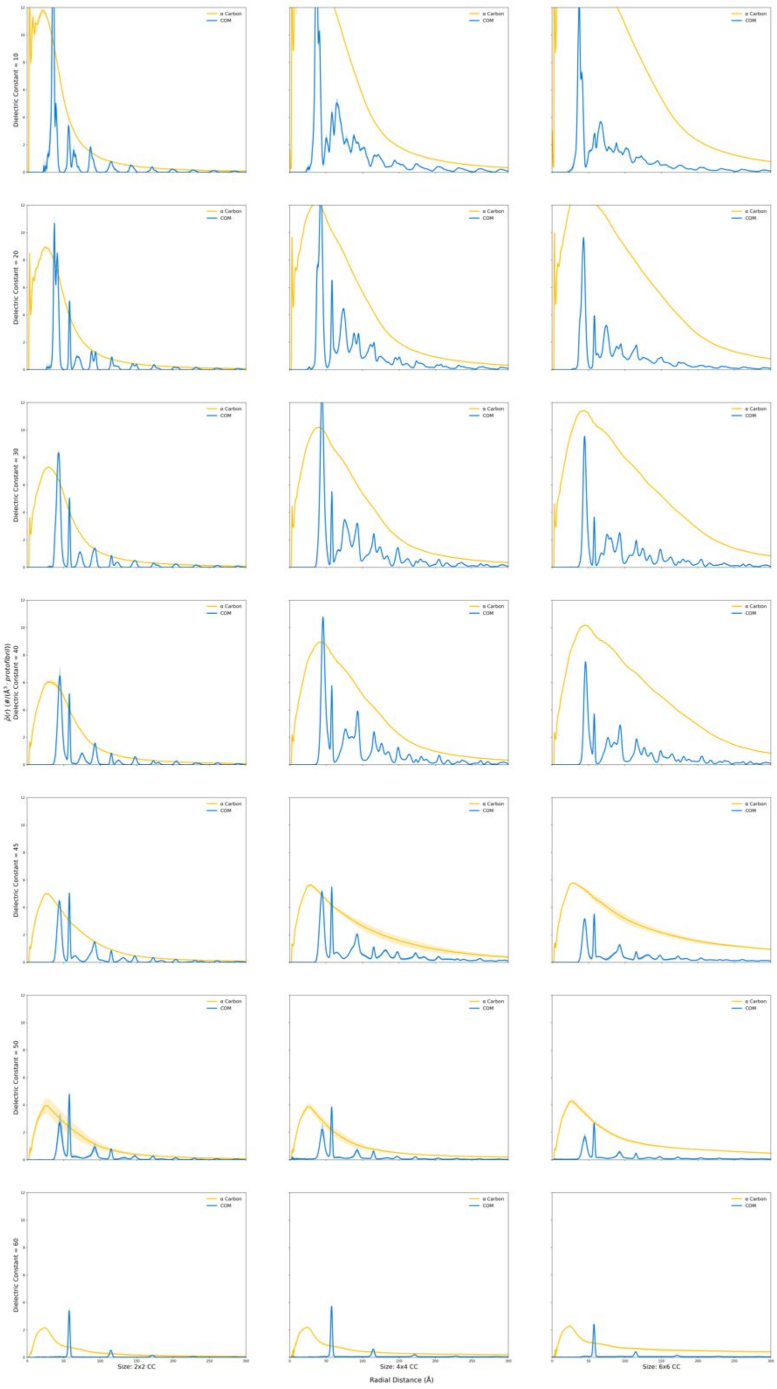


**Figure S17**. Radial density distributions for all CG MD fibril simulations.

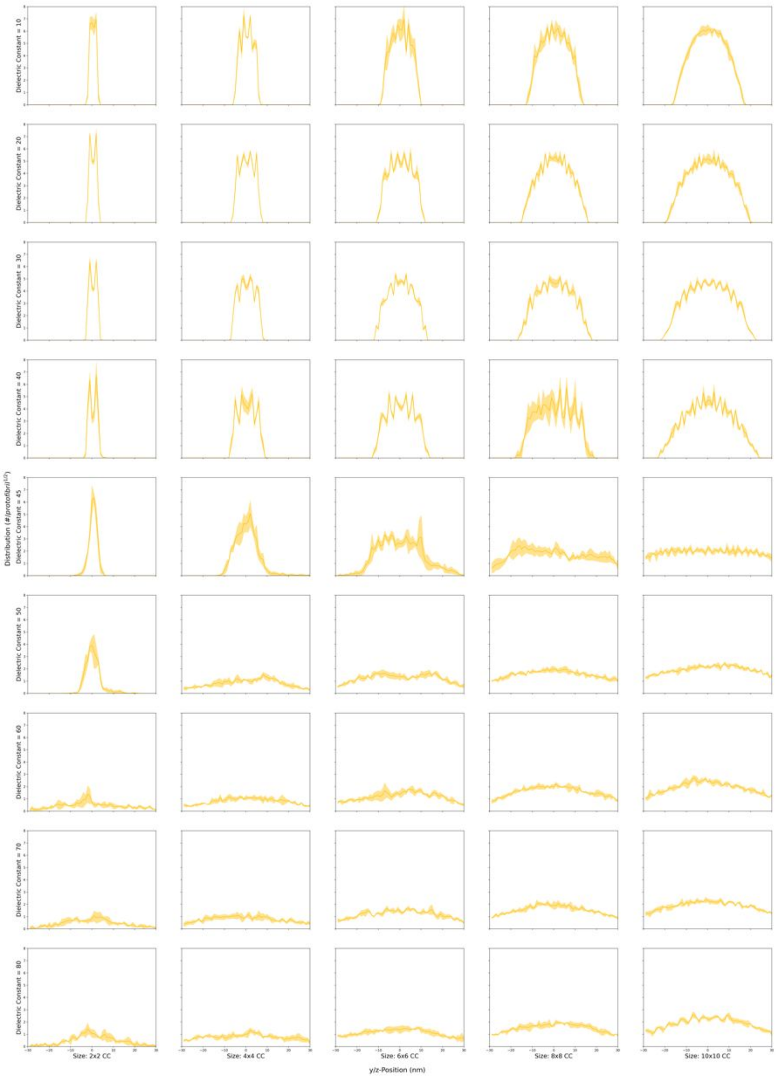


**Figure S18**. The normalized number density from each CG MD fibril simulation used to calculate the diameter of fibrils in **Figure 6c**.

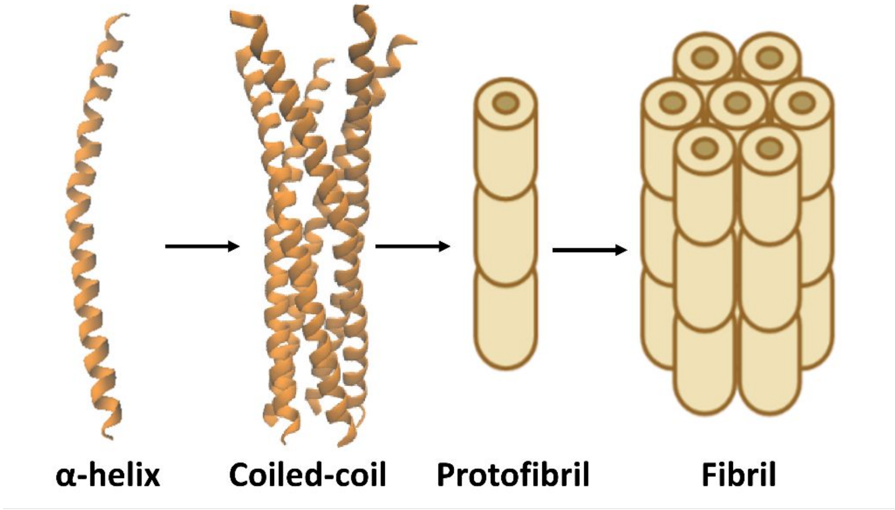


**Figure S19**. The expected hierarchy of assembly for Q5. Here, Q5 chains (monomers) assemble into a pentameric coiled-coil, which subsequently stack with other coiled-coils end-to-end to form protofibrils. The protofibrils align in parallel to form a complete fibril. The initial structures for each CG fibril were constructed following this hierarchy.

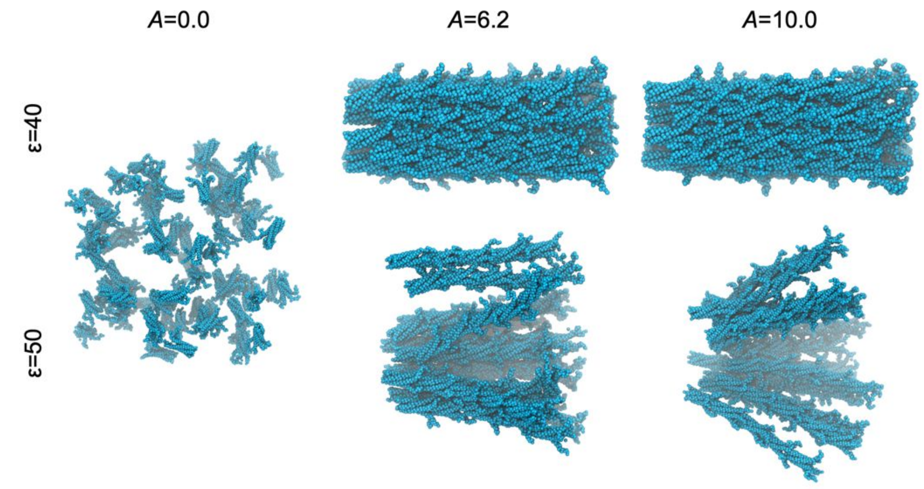


**Figure S20**. CGMD simulations of a 4 CC by 4 CC fibril consisting of protofibrils of length 14 CCs using a range of Gaussian prefactor parameters (*A*, in kcal/mol) and dielectric constants (ε).

**Table S1** Critical gelation time, $t_c$, and critical gelation exponent, nc calculated using MPT and superposition analysis and storage modulus (G') and loss modulus (G'') at 10 Hz calculated by parallel plate rhinometry for Q3-7 hydrogel variants.

| **Protein** | ***$t_c$* (h)** | ***$n_c$*** | **G' (Pa)** | **G'' (Pa)** |
|---|---|---|---|---|
| **Q3** | 58 ± 0.4 | 0.52 ± 0.01 | 82.5 ± 35.4 | 12.3 ± 1.7 |
| **Q4** | 21.6 ± 2.0 | 0.56 ± 0.03 | 69.9 ± 19.7 | 15.0 ± 3.2 |
| **Q5** | 11.5 ± 1.5 | 0.49 ± 0.02 | 228.7 ± 52.2 | 11.8 ± 6.1 |
| **Q6** | 48.3 ± 1.7 | 0.60 ± 0.03 | 252.9 ± 122.7 | 13.0 ± 5.7 |
| **Q7** | 37.1 ± 0.1 | 0.58 ± 0.0 | 297.5 ± 61.7 | 20.6 ± 7.9 |

**Table S2** Equation fit averages for hydrogel variant FRAP experiment using one-phase association equation in GraphPad Prism.

| **Protein** | **Y0 (pixel$^{-1}$)** | **Plateau (pixel$^{-1}$)** | **K (s$^{-1}$)** | **Tau (s)** | **Halftime (s)** | **Span (pixel$^{-1}$)** |
|---|---|---|---|---|---|---|
| **Q** | 14.5 ± 0.2 | 19.6 ± 0.3 | 0.1 ± 0.0 | 14.3 ± 0.2 | 9.9 ± 0.1 | 5.1 ± 0.2 |
| **Q2** | 29.4 ± 1.6 | 41.8 ± 2.8 | 0.1 ± 0.0 | 16.0 ± 0.6 | 11.1 ± 0.4 | 12.5 ± 1.3 |

| | | | | | | |
|---|---|---|---|---|---|---|
| Q3 | 8.5 ± 3.9 | 14.4 ± 7.5 | 0.0 ± 0.1 | 50.1 ± 45.2 | 34.8 ± 31.3 | 5.9 ± 3.6 |
| Q4 | 45.1 ± 0.2 | 56.2 ± 0.2 | 0.1 ± 0.0 | 8.2 ± 0.5 | 5.7 ± 0.3 | 11.1 ± 0.2 |
| Q5 | 5.3 ± 0.0 | 6.4 ± 0.0 | 0.1 ± 0.0 | 17.7 ± 1.3 | 12.3 ± 0.9 | 1.2 ± 0.0 |
| Q6 | 34.1 ± 4.8 | 39.1 ± 5.6 | 0.1 ± 0.0 | 7.3 ± 2.5 | 5.1 ± 1.7 | 5.1 ± 0.8 |
| Q7 | 7.05 ± 0.0 | 10.0 ± 0.0 | 0.1 ± 0.0 | 13.9 ± 0.3 | 9.7 ± 0.2 | 3.0 ± 0.0 |

**Table S3** Mean residue ellipticity (MRE) of minima at 222 nm and 208 nm from CD spectra. Results represent the average of three independent trials.

| **Protein** | **Solution/Gel State** | $\boldsymbol{\theta_{222}}$ **(mdeg•cm²•dmol⁻¹)** | $\boldsymbol{\theta_{208}}$ **(mdeg•cm²•dmol⁻¹)** | $\boldsymbol{\theta_{222}/\theta_{208}}$ |
|---|---|---|---|---|
| Q3 | Solution | -3,000 ± 1,000 | -2,000 ± 1,000 | 1.2 ± 0.1 |
| | Gel | -4,000 ± 1,000 | -2,000 ± 0 | 2.3 ± 0.3 |
| Q4 | Solution | -7,000 ± 1,000 | -6,000 ± 1,000 | 1.3 ± 0.3 |
| | Gel | -5,000 ± 1,000 | -5,000 ± 1,000 | 1.4 ± 0.2 |
| Q5 | Solution | -18,000 ± 1,000 | -16,000 ± 2,000 | 1.2 ± 0.1 |

| | | | | |
|---|---|---|---|---|
| | Gel | -8,000 ± 2,000 | -5,000 ± 1,000 | 1.6 ± 0.1 |
| Q6 | Solution | -27,000 ± 6,000 | -25,000 ± 6,000 | 1.1 ± 0.0 |
| | Gel | -16,000 ± 1,000 | -16,000 ± 1,000 | 1.5 ± 0.1 |
| Q7 | Solution | -15,000 ± 1,000 | -16,000 ± 1,000 | 1.0 ± 0.0 |
| | Gel | -15,000 ± 1,000 | -16,000 ± 2,000 | 1.2 ± 0.1 |

**Table S4** ATR-FTIR compositional analysis from Q3-7 protein in solution and gel states. Summary of secondary structure content uses the average and standard deviation of the integrated area of deconvoluted peaks from three independent trials.

| Q Hydrogel Variant | Solution/Gel State | α-helix (%) | β-sheet(%) | Turns/Coils (%) |
|---|---|---|---|---|
| Q3 | Solution | 32.1 ± 6.1 | 46.9 ± 4.2 | 21.0 ± 3.5 |
| | Gel | 34.1 ± 6.1 | 46.5 ± 3.8 | 19.3 ± 3.2 |
| Q4 | Solution | 42.2 ± 8.8 | 45.2 ± 7.2 | 15.2 ± 9.4 |
| | Gel | 34.7 ± 6.0 | 48.6 ± 4.2 | 16.8 ± 3.5 |
| Q5 | Solution | 39.8 ± 0.7 | 44.2 ± 0.2 | 16.1 ± 0.6 |

| | | | | |
|---|---|---|---|---|
| | Gel | 41.2 ± 3.8 | 48.6 ± 6.3 | 10.0 ± 8.7 |
| **Q6** | Solution | 27.4 ± 6.8 | 57.6 ± 2.1 | 26.7 ± 7.6 |
| | Gel | 33.8 ± 1.6 | 46.9 ± 2.6 | 19.3 ± 2.5 |
| **Q7** | Solution | 27.8 ± 7.8 | 40.6 ± 16.0 | 27.0 ± 8.5 |
| | Gel | 32.9 ± 6.3 | 37.6 ± 4.4 | 29.4 ± 6.8 |

**Table S5**. The Alphafold2-Multimer[11] pLDDT confidence metric for different Q5 coiled-coil multimers based on the highest ranked multimeric structure (out of 5 predictions).

| System | Confidence |
|---|---|
| 4-mer | 0.7165 |
| 5-mer | 0.7364 |
| 6-mer | 0.6741 |

## SI REFERENCES


(1) Hill, L. K.; Meleties, M.; Xie, X.; Delgado-Fukushima, E.; Jihad, T.; Liu, C. F.; O'Neill, S.; Tu, R. S.; Renfrew, P. D.; Bonneau, R.; et al. Thermoresponsive Protein-Engineered Coiled-coil Hydrogel for Sustained Small Molecule Release. *Biomacromolecues* **2019**, *20*, 3340-3351. DOI: https://doi.org/10.1021/acs.biomac.9b00107.

(2) Britton, D.; Meleties, M.; Liu, C.; Jia, S.; Mahmoudinobar, F.; Renfrew, P. D.; Bonneau, R.; Montclare, J. K. Tuning a Coiled-coil Hydrogel via Computational Design of Supramolecular Fiber Assembly. *Molecular Systems Design & Engineering* **2022**, 10.1039/D2ME00153E. DOI: 10.1039/D2ME00153E.

(3) Meleties, M.; Britton, D.; Katyal, P.; Lin, B.; Martineau, R. L.; Gupta, M. K.; Montclare, J. K. High-Throughput Microrheology for the Assessment of Protein Gelation Kinetics. *Macromolecules* **2022**, *55* (4), 1239-1247. DOI: 10.1021/acs.macromol.1c02281.

(4) Gunasekar, S. K.; Asnani, M.; Limbad, C.; Haghpanah, J. S.; Hom, W.; Barra, H.; Nanda, S.; Lu, M.; Montclare, J. K. N-Terminal Aliphatic Residues Dictate the Structure, Stability, Assembly, and Small Molecule Binding of the Coiled-Coil Region of Cartilage Oligomeric Matrix Protein. *Biochemistry* **2009**, *48* (36), 8559-8567. DOI: 10.1021/bi900534r.

(5) Jackson, M.; Mantsch, H. H. The use and misuse of FTIR spectroscopy in the determination of protein structure. *Crit Rev Biochem Mol Biol* **1995**, *30* (2), 95-120. DOI: 10.3109/10409239509085140 From NLM.

(6) Wang, P.; Bohr, W.; Otto, M.; Danzer, K. M.; Mizaikoff, B. Quantifying amyloid fibrils in protein mixtures via infrared attenuated-total-reflection spectroscopy. *Analytical and Bioanalytical Chemistry* **2015**, *407* (14), 4015-4021. DOI: 10.1007/s00216-015-8623-4.

(7) Hu, X.; Kaplan, D.; Cebe, P. Determining Beta-Sheet Crystallinity in Fibrous Proteins by Thermal Analysis and Infrared Spectroscopy. *Macromolecules* **2006**, *39* (18), 6161-6170. DOI: 10.1021/ma0610109.

(8) Schneider, C. A.; Rasband, W. S.; Eliceiri, K. W. NIH Image to ImageJ: 25 years of image analysis. *Nat Methods* **2012**, *9* (7), 671-675. DOI: 10.1038/nmeth.2089 From NLM.

(9) Riback, J. A.; Bowman, M. A.; Zmyslowski, A. M.; Knoverek, C. R.; Jumper, J. M.; Hinshaw, J. R.; Kaye, E. B.; Freed, K. F.; Clark, P. L.; Sosnick, T. R. Innovative scattering analysis shows that hydrophobic disordered proteins are expanded in water. *Science* **2017**, *358* (6360), 238-241. DOI: 10.1126/science.aan5774 (acccessed 2023/04/03).

(10) Riback, J. A.; Bowman, M. A.; Zmyslowski, A. M.; Plaxco, K. W.; Clark, P. L.; Sosnick, T. R. Commonly used FRET fluorophores promote collapse of an otherwise disordered protein. *Proceedings of the National Academy of Sciences* **2019**, *116* (18), 8889-8894. DOI: 10.1073/pnas.1813038116 (acccessed 2023/04/04).

(11) Evans, R.; O'Neill, M.; Pritzel, A.; Antropova, N.; Senior, A.; Green, T.; Žídek, A.; Bates, R.; Blackwell, S.; Yim, J.; et al. Protein complex prediction with AlphaFold-Multimer. *BioRxiv* **2022**. DOI: doi: https://doi.org/10.1101/2021.10.04.463034.
